# Dual PI3K and HDAC inhibitor, CUDC-907, effectively inhibits endometrial cancer growth *in vitro* and *in vivo*

**DOI:** 10.3389/fonc.2025.1531805

**Published:** 2025-11-04

**Authors:** Xudong Zhang, Vanessa Lazaro-Camp, Tianyue Li, Amanda Qi, Lingyun Lan, Lillie Lamont, Lu Zhao, Maggie R. Meehan, Sophia N. Gardner, Wendy Meng, Yiqin Xiong, Mariah Leidinger, Yumi Imai, Xiangbing Meng, Shujie Yang

**Affiliations:** ^1^ Department of Pathology, Carver College of Medicine, University of Iowa, Iowa City, IA, United States; ^2^ Department of Internal Medicine, Carver College of Medicine, University of Iowa, Iowa City, IA, United States; ^3^ Department of Ophthalmology, Carver College of Medicine, University of Iowa, Iowa City, IA, United States; ^4^ Holden Comprehensive Cancer Center, University of Iowa, Iowa City, IA, United States

**Keywords:** CUDC-907, endometrial cancer, PI3K/AKT, HDAC, progestin therapy, progesterone receptor, IGF-1

## Abstract

**Purpose:**

Hyperactivation of the PI3K/AKT/mTOR pathway promotes tumor progression in many cancers. Among these, endometrial cancer (EC) exhibits the highest frequency of alterations in this pathway, making it an ideal model for targeted treatment. Progestin therapy is initially effective, but advanced EC often resists treatment due to loss of progesterone receptor (PR) and acquired resistance. Furthermore, as obesity is the main etiological driver of EC, obesity-related factors activate the PI3K/AKT pathway and inhibit PR function. Therefore, there is a clinical need to identify therapies that enhance progestin sensitivity by upregulating PR, downregulating obesity-related factors, and inhibiting the PI3K/AKT pathway.

**Methods:**

A dual HDAC (histone deacetylase) and PI3K inhibitor, CUDC-907 (fimepinostat), was tested for its ability to inhibit the proliferation of endometrial cancer cells both *in vitro* and *in vivo* by targeting PI3K and HDAC pathways. A WST-1 Cell Proliferation Colorimetric Assay Kit was used to assess cell viability. Western blotting was used for protein expression. Endometrial cancer xenograft models were established in mice fed a high-fat-diet, normal chow, or subjected to fasting to evaluate the drug’s activity under different metabolic conditions. Serum biomarkers were quantified using enzyme-linked immunosorbent assay (ELISA).

**Results:**

Rapid inhibition of the PI3K/AKT pathway was observed; CUDC-907 treatment downregulated p-AKT, p-rS6, and p-4EBP1. Concurrently, transcriptional inhibition of HDAC activity was also observed. PR expression was restored, downstream genes FOXO1, p21, and H3Ace were upregulated, and oncogenes Myc and HER2 (Neu/ErbB2) were downregulated. CUDC-907 induced both intrinsic and extrinsic apoptotic pathways. *In vivo*, CUDC-907 inhibited EC progression, increased survival of tumor-bearing mice, and suppressed tumor growth. Notably, CUDC-907 was most effective in reducing tumor growth in mice on high-fat diets. Furthermore, serum IGF-1 levels decreased following CUDC-907 treatment, suggesting that IGF-1 may serve as a surrogate serum marker for the CUDC-907 drug’s effect in EC.

**Conclusion:**

Our findings suggest that CUDC-907 is a promising agent that can re-sensitize tumors to progestin therapy and improve outcomes for EC patients. This study supports CUDC-907 as a potent treatment strategy in endometrial cancer and identifies IGF-1 as a potential surrogate serum biomarker for therapeutic response.

## Introduction

In 2025, it is estimated that there will be 69,120 new cases of endometrial cancer (EC) and 13,860 related deaths in the United States (1), highlighting the critical need for improved interventions. EC is the most common gynecologic maligancy and primarily affects postmenopausal women, with rising incidence and mortality. EC is the only major cancer type in the US with a declining survival rate over the past 40 years (2). This underscores the urgency of developing effective therapies for EC, which remains a major health concern. This disease is unique in three aspects.

First, EC is highly sensitive to hormonal regulation, particularly to the growth-inhibitory effects of progesterone, which has been described as the ultimate endometrial tumor suppressor (3). Progestin therapy has demonstrated promising clinical outcomes in PR (progesterone receptor)-positive tumors. However, many aggressive ECs lose PR expression, which is associated with poorer outcomes and resistance to progestin therapy. Therefore, restoring or upregulating PR expression is essential to re-sensitize tumors to progestin therapy and improve patient outcomes.

Second, obesity has a stronger association with EC than with any other cancer type (4, 5). Obesity-related factors such as IGF-I activates the PI3K/Akt pathway and suppress PR funciton, contributing to EC progression (6, 7). Inhibition of the PI3K/Akt pathway in EC has been shown to restore PR expression, re-sensitize tumors to progestin therapy, and reduce angiogenesis (8–10).

Third, the PI3K/Akt signaling pathway is hyperactivated in many cancers, particularly in EC. Over 90% of ECs exhibit genomic alteration of the PI3K/Akt pathway (6), due to two main factors: (1) direct activation by the aforementioned obesity-related factors and (2) the high frequency of mutations in *PIK3CA*, *PIK3R1*, and *PTEN* found in EC. Specifically, PI3K/Akt pathway activation has been observed across all four genomic subtypes of EC: POLE ultramutated (94%), microsatellite instability hypermutated (MSI) (95%), copy-number low (90%), and copy-number high (60%). Furthermore, a 2013 study assessing twenty signaling pathways across twelve major cancer types found that 52.2% of EC samples harbored PIK3CA mutations, 30.9% had PIK3R1 mutations, and 63.5% had PTEN mutations (11). These data indicate that targeting the PI3K/Akt pathway is a compelling therapeutic strategy in EC. However, therapies that target this pathway alone rarely produce durable responses due limited selectivity, compensatory signaling loops, feedback mechanisms, and (epi)genetic alterations. Additionally, resistance mechanisms have been reported, including PI3K-dependent feedback (12), FOXO-mediated feedback loops, and Myc-dependent resistance (13, 14).

In addition to the PI3K/Akt/mTOR pathway inhibitors, histone deacetylase (HDAC) inhibitors have also been widely explored in cancer treatment. HDACs regulate transcription and the acetylation of various cellular substrates, thereby contributing to cancer initiation and progression (15). Our previous study demonstrated that multiple HDAC inhibitors, including LBH589, SAHA, PXD101, and romidepsin can restore functional PR expression and induce G1 cell cycle arrest in EC (16–18). Based on these findings, the oral HDAC inhibitor entinostat was selected for the surgical-window clinical trial GY011 (18, 19). This clinical trial was designed for newly diagnosed EC patients to evaluate whether entinostat could restore PR expression and activity. Although entinostat led to reduced expression of the proliferation marker Ki67, it did not restore PR expression (19). Other studies have also shown that, as single therapeutic agents, HDAC inhibitors have limited therapeutic efficacy against solid tumors in clinical settings (20) (21).

While monotherapy with HDAC inhibitors provides only modest benefit, combining PI3K inhibitors with HDAC inhibitors has been shown to enhance therapeutic efficacy, as suggested by previously reported synergistic effects (22). CUDC-907 (fimepinostat), a dual HDAC/PI3K inhibitor, has shown promising activity in multiple cancer types, but its efficacy in EC has not been previously reported.

Progesterone or synthetic progestins are commonly used in conservative treatment regimens for EC patients who are either ineligible for or unwilling to undergo hysterectomy. However, responsiveness to these treatments diminishes over time due to the progressive loss of PR expression, and recurrences are common. Therefore, in addition to evaluating the efficacy of CUDC-907 alone, we assessed the drug in combination with progestin to determine whether CUDC-907 treatment can resensitize tumors to progestin therapy.

Our findings demonstrate that CUDC-907 successfully resensitizes tumors to progestin therapy by upregulating PR expression, downregulating the obesity-related factor IGF-1, and inhibiting the PI3K/Akt/mTOR signaling pathway. Ultimately, CUDC-907 suppressed tumor growth and prolonged survival in treated mice. Therefore, CUDC-907 represents a promising treatment strategy for EC, as it effectively targets three key drivers of EC disease progression.

## Materials and methods

### Reagents

CUDC-907 was provided by Curis, Inc. Panobinostat (LBH589) was purchased from Selleck Chemicals. BKM120 was provided by Novartis. Medroxyprogesterone acetate (MPA, USP, 150mg/ml) was purchased from Pfizer (New York, NY). For *in vitro* assays, CUDC-907 was dissolved in dimethyl sulfoxide (DMSO). Stock solutions (50mmol/L) were prepared and stored at -80°C for single use. For *in vivo* studies, CUDC-907 was formulated in 30% Captisol (Ligand Pharmaceuticals, Inc.) according to Curis formulation guidelines.

### Cell lines and culture

EC cell lines ECC1 and KLE were purchased from ATCC and were cultured according to ATCC guidelines. Ishikawa H and Hec50 cells were kindly provided by Dr. Erlio Gurpide (New York University) and maintained in DMEM supplemented with 10% FBS (Gibco). All cell lines were authenticated using short tandem repeat (STR) analysis performed by BioSynthesis.

### IC_50_ determination for BKM120, LBH589, and CUDC 907

Cells were seeded at 10,000 cells per well in 96-well plates After 24 hours, cells were treated at approximately 30% confluency and incubated for 72 hours. BKM120 was tested at concentrations from 0.1nM to 1μM. LBH589 and CUDC-907 were tested at concentration from 0.1nM to 100nM. Cell proliferation was assessed using the WST-1 assay (MK400, TaKaRa) and IC_50_ values were determined accordingly.

### Synergistic effect analysis

Ishikawa cells were seeded at 10,000 cells per well in 96-well plates (After 24 hours, cells were treated at approximately 30% confluency and incubated for 72 hours. MPA or progesterone was tested from at concentration ranging from 1µM to 20μM, and CUDC 907 was tested at concentrations from 0.1nM to 100nM. Cell proliferation was assessed using the resazurin assay (#7017, Sigma), and synergistic effects were analyzed using SynergyFinder (https://synergyfinder.fimm.fi).

### Western blotting

Protein extraction and quantification from cultured cells were conducted as previously described (17, 18). Protein from snap-frozen tumor tissue lysates was extracted using RIPA lysis buffer (1% NP-40, 150mM NaCl, 50mMTris HCl, 0.5% Sodium Deoxycholate, 0.1% SDS, pH 7.2) and quantified using the DC Protein Assay Kit II (Bio-Rad, 5000112). The following antibodies were purchased from Cell Signaling Technology (Danvers, MA, USA): PR (#3153 and #3157), AKT (#9272), phospho‐AKT (Ser473, #4060), FOXO1(#2880), H3Ace (#39139), Myc (#13987), PARP (#9542), Caspase3 (#9662), and Caspase8 (#9746). Survivin (sc17779) and Her2 (Neu, sc33864) were obtained from Santa Cruz Biotechnology (Dallas, TX, USA). β‐actin (A1978) was purchased from Sigma and used as a loading control.

### *In vivo* tumor models and efficacy studies

Six- to nine-week-old female immunodeficient athymic (NOD.Cg-Prkdc<scid>, 005557) mice were obtained from The Jackson Laboratory. Mice were subcutaneously injected with 1 x 10^6^ Ishikawa cells suspended in 200 µL of medium into the right and left flank regions. The following week, all mice were ovariectomized. Once the average tumor size reached approximately 100 mm^3^, mice were randomized into four groups: (1) control, (2) MPA, (3) CUDC-907, and (4) MPA+CUDC-907. Groups 2 and 4 received 100 μL of 10mg/ml MPA (equivalent to1mg), administered intramuscularly once per week. CUDC-907 was administered orally on Monday, Wednesday, and Friday for over two weeks. Tumor size was measured three times per week, and volume was expressed in mm^3^ using the formula, V = 0.5ab^2^, where a and b were the long and short diameters of the tumor, respectively. Tumor volumes were used to calculate tumor growth inhibition (TGI), using the formula: TGI=[(1-T/C) *100], where C is the average tumor volume of control mice, and T is the average tumor volume of treated mice. The larger tumor from each mouse was used for TGI calculation to reflect clinical practice.

Sample size justification: Based on power calculations to detect >30% difference in tumor volume (SD = 20%, α=0.05, power=80%), the estimated minimum sample size was 6–8 mice per group. To account for biological variability and animal loss, 6–10 mice were used per group. The calculation was based on a two-sample t-test, consistent with preclinical efficacy study standards.

### Survival study

Groups 2 and 4 received MPA, while group 3 and 4 received CUDC-907 as described above. The mice were treated until tumor volume reached 2,000mm^3^ or the long diameter reached 2 cm. Mice were euthanized if they appeared severely ill (e.g., reduced spontaneous activity, unkempt coat, and dehydrated appearance), lost> 20% body weight or showed signs of limb paralysis. Blood was drawn from the tail vein, allowed to settle for 15–30 minutes, and then centrifuged at 1,000 rpm for 15 minutes. Serum was collected and saved at -80°C.

### ELISA

A mouse obesity ELISA kit was purchased from Signosis and included assays for Leptin, TNFα, IGF-1, IL-6, Insulin, IL-1α, IL-1β, and MCP-1. Mouse IGF-1 ELISA Kit was purchased from Signosis and R&D Systems. Serum was diluted 1:10 in PBS, and IGF-1 levels were measured according to the manufacturer’s instructions.

### Quantitative PCR experiments

RNA was purified from cell lysates using RNeasy columns (Qiagen). cDNA was synthesized using the ProtoScript III First-Strand Synthesis kit for qRT-PCR (New England BioLabs) Quantitative PCR (qPCR) was performed in triplicate using SYBR Green PCR Master Mix and run on an Applied Biosystems instrument Relative gene expression was normalized to 18s rRNA and calculated using the ΔΔCt fold change method. Primer sequences are listed in **Table 1**.

**Table 1:** Primer sequences used for quantitative RT-PCR analysis.

	Gene	Sequence
1	SCGB1D2-F	AGTGAAGAGATGCACGGATCA
2	SCGB1D2-R	CACCAGGACTTCCGCAATGA
3	FKBP5-F	CTCCCTAAAATTCCCTCGAATGC
4	FKBP5-R	CCCTCTCCTTTCCGTTTGGTT
5	CD52-F	TCTTCCTCCTACTCACCATCAG
6	CD52-R	CCTCCGCTTATGTTGCTGGA
7	DLK1-F	AGGGTCCCCTTTGTGACCA
8	DLK1-R	GCAGGCCCGAACATCTCTATC
9	UNC5C-F	ACCTGTACTGTAAAGCAAGCC
10	UNC5C-R	GGACAATGAGACCGGAAGTTT
11	GALNT9-F	GGAAGCCCTACAACAACGACA
12	GALNT9-R	GGGTTCGACATGGGGATGTTC
13	GNG2-F	CTCAGGCTTTAGGAACTGAAGAG
14	GNG2-R	GCTGCCTTGGACACCTTTAT
15	SCUBE1-F	TGGACGAGTGTCAGGACAATA
16	SCUBE1-R	GCAGTTCATACCCTCATTGGAG
17	GDNF-F	GGCAGTGCTTCCTAGAAGAGA
18	GDNF-R	AAGACACAACCCCGGTTTTTG
19	TMEM35A-F	CCCAGAACCGTAACTATTGTGG
20	TMEM35A-R	GTTTCATCTCACTGTAGGCATCC

List of forward (F) and reverse (R) primer sequences used to validate differentially expressed genes identified from RNA sequencing of CUD-907-treated Ishikawa tumors. Primer sequences were designed using NCBI Primer-BLAST and synthesized by Integrated DNA Technologies (IDT).

### H&E staining and IHC

Mouse tumor tissues were fixed in formalin, routinely processed, paraffin-embedded, and sectioned at 4 µm thick. After mounting the tissues on microscope slides, H&E staining or immunohistochemistry (IHC) staining was performed by the Histology Core Laboratory in the Department of Pathology. Slides were dewaxed and rehydrated with successive washed in xylene, 100% ethanol, 70% ethanol, and tap water. Hematoxylin was applied for 4 minutes, followed by 20 seconds of differentiation in ammonia water, then eosin was applied for 20 seconds. The following primary antibodies were used for IHC: p21 (ab109520) and Ki67 (#16667 clone SP6) from Abcam, p-Akt (#4060) Cell Signaling, H3Ace (#39139) antibody from Active Motif, PR (M3568) from Dako.

### Transcriptome analysis

Ishikawa tumor-bearing mice were treated with no treatment control, 1mg/mouse of MPA, 100 mg/kg of CUDC-907, or MPA + CUDC for 24 hours. One hour prior to sacrifice, mice in the CUDC-907 and MPA+CUDC-907 groups received an additional dose of 100mg/kg of CUDC-907. Tumors were collected for RNA-Seq. Total RNA was purified as mentioned above.

Sequencing was performed on an Illumina NovaSeq 6000 System (Arraystar). RNA-Seq data were analyzed using the Bioconductor package Limma. RNA-seq counts from 12 samples (control = 3, MPA = 3, CUDC-907 = 3, and MPA+CUDC-907 = 3) were pre-processed prior to differential expression analysis.

### Statistical analysis

All data are displayed as mean ± SEM. A one-way ANOVA and a two-sample student t-test assuming unequal variances (one-tailed) were used for comparison. Statistical analysis was conducted using Microsoft Excel. Confidence intervals (CIs) were calculated using this formula: CI = Mean ± (1.96 x SEM). Differences between tumor values under different experimental conditions were evaluated using the Student’s t-test. A *p-value* < 0.05 was considered statistically significant.

For drug combination studies, synergy analysis was performed using the SynergyFinder web tool (https://synergyfinder.fimm.fi). The Zero Interaction Potency (ZIP) model was applied to assess the interaction between CUDC-907 and MPA, as well as CUDC-907 and progesterone (P4). Both the average synergy score and the most synergistic area score were reported to evaluate overall and localized synergistic effects, respectively. According to the ZIP model, synergy scores between -10 and +10 indicates an additive effect, a score greater than +10 suggest synergism, while a score less than -10 indicates antagonism.

## Results

### CUDC-907 inhibits endometrial cancer growth *in vitro* via inhibition of HDAC activity and the PI3K/Akt pathway

**Figure 1:**
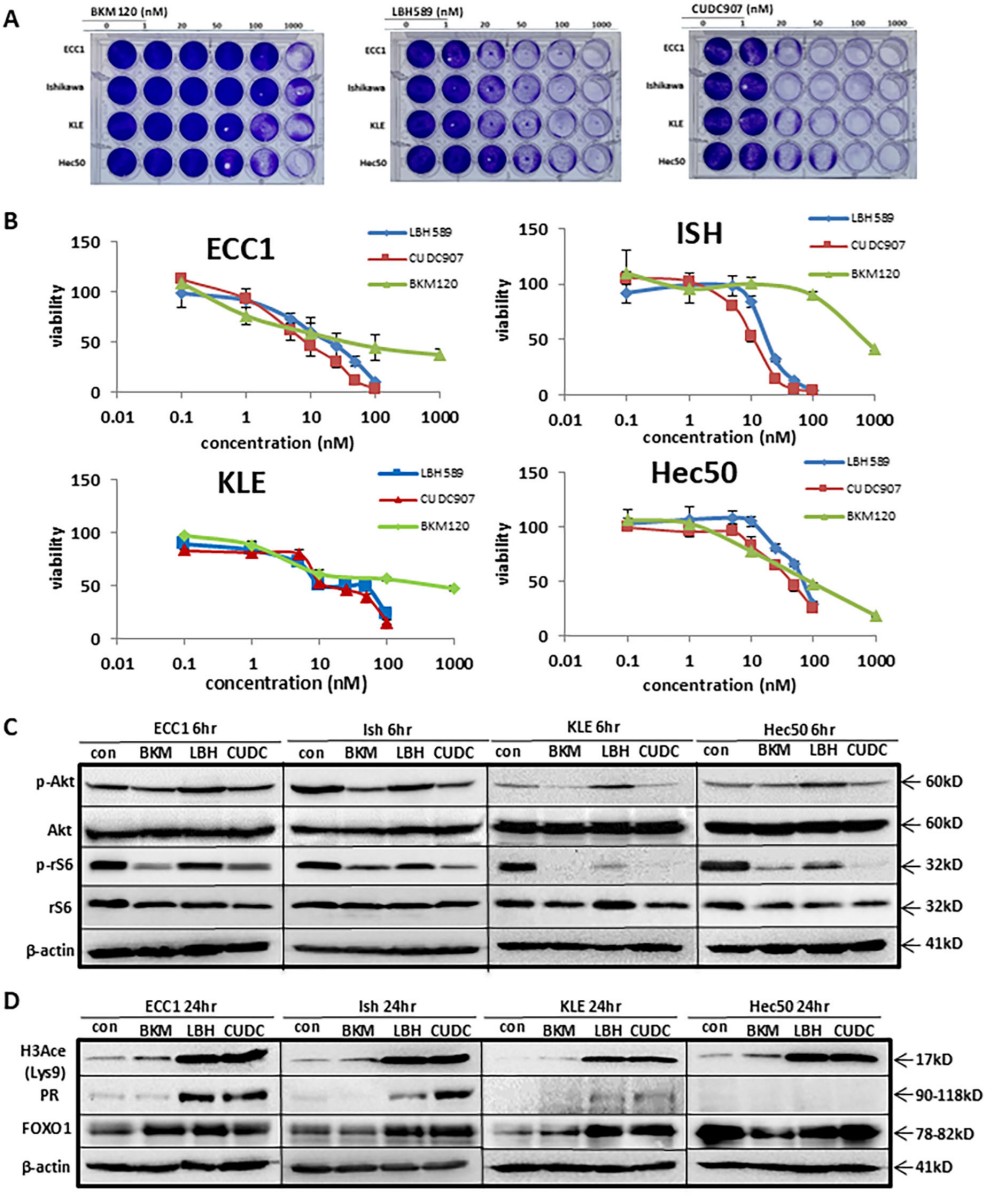
CUDC-907 was confirmed to inhibit PI3K and HDAC in EC cells. **(A)** Four EC cells were treated with BKM120, LBH589, and CUDC-907 at the indicated dose for 72hr. Cell viability was assessed via crystal violet. **(B)** IC50 assay was conducted to assess cell viability in all four cell lines after 72hr treatment with BKM120, LBH589, and CUDC-907. **(C, D)** Western blotting was conducted over 6hr and 24hr time courses in all four cell lines following treatment with BKM120, LBH589, and CUDC-907 at the indicated concentrations.

To evaluate the growth-inhibitory effect of CUDC-907 in EC, four EC cell lines (ECC1, Ishikawa, KLE, and Hec50) were treated with a PI3K inhibitor (BKM120), an HDAC inhibitor (LBH589), or the dual PI3K/HDAC inhibitor CUDC-907. CUDC-907 induced dose-dependent cytotoxicity in all four cell lines, with varying sensitivity (**Figure 1A**). BKM120 exhibited the least cytotoxicity, with significant reduction in cell proliferation observed only in Hec50 at 1,000 nM. LBH589 was more effective than BKM120, with decreased proliferation at 20nM for ECC1, Ishikawa, and Hec50 cells. Importantly, CUDC-907 demonstrated the greatest efficacy, significantly inhibiting proliferation in all four cell lines at concentration as low as 20nM.

As shown in **Figure 1B** and **Supplementary Table 1**, IC_50_ assays were performed to quantify the effects on cell proliferation across the four EC cell lines. BKM120 inhibited cell proliferation at 15nM in ECC1, 75nM in Hec50 cells, 650nM in Ishikawa and 250nM in KLE cells. In contrast, LBH589 and CUDC-907 were more potent, inhibiting ECC1 and Ishikawa cell proliferation at <20nM, and KLE and Hec50 cell proliferation at approximately 25-100nM. ECC1 and Ishikawa cell lines were more sensitive to CUDC-907 treatment than KLE or Hec50 cells.

To assess the dual activity of CUDC-907, we compared its effect to mono-inhibitors targeting either PI3K or HDAC. As PI3K inhibitors, both CUDC-907 and BKM120 effectively reduced p-Akt (Ser473) levels in Ishikawa, KLE, and ECC1 cells, more effectively than LBH589. Additionally, p-rS6 was inhibited by both CUDC-907 and BKM120 in all four cell lines, whereas LBH589 inhibited p-rS6 only in KLE and Hec50 cells. CUDC-907 showed superior potency in suppressing p-rS6 in Hec50 cells (**Figure 1C**).

As HDAC inhibitors, both CUDC-907 and LBH589 significantly upregulated acetylated H3 (H3Ace) (**Figure 1D**). Both drugs also increased PR expression in ECC1, Ishikawa, and KLE cells, although CUDC-907 induced stronger PR expression in Ishikawa cells. In Hec50 cells, PR remained undetectable following treatment with either drug, potentially due to permanent PR repression mediated by DNA methylation, as previously reported by our group (23) (**Figure 1C-D**). Together, these findings confirm that CUDC-907 effectively targets both the PI3K/Akt signaling pathway and HDAC activity, resulting in enhanced growth inhibition and PR restoration in endometrial cancer cells.

### CUDC-907 inhibits the PI3K/Akt pathway and HDAC activity, exhibiting broader antitumor effects

**Figure 2:**
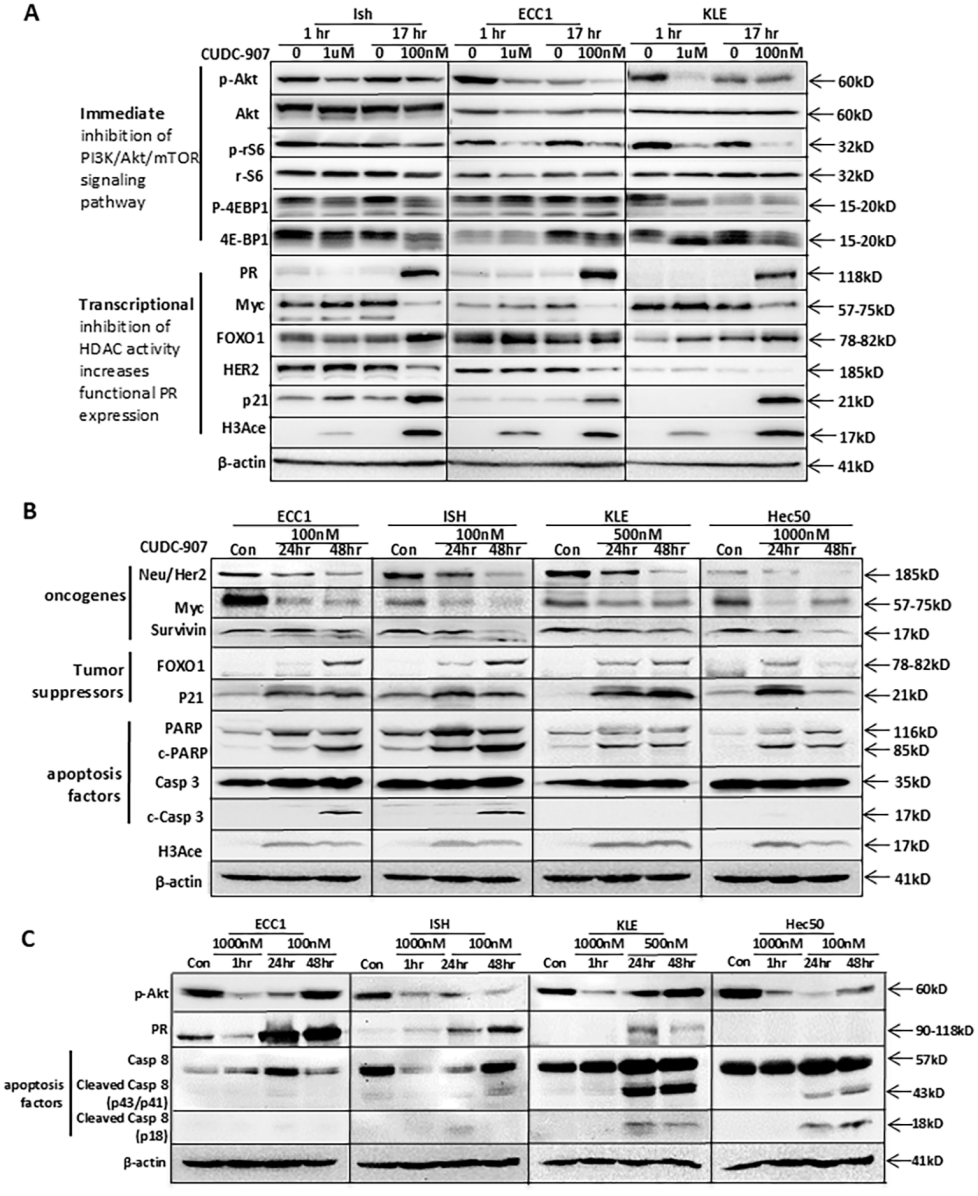
CUDC-907 immediately inhibits the PI3K/Akt pathway, leads to transcriptional inhibition of HDAC activity, and has broader antitumor effects. Multiple EC cell lines were tested at the indicated dose and time course. **(A)** PI3K/Akt/mTOR downstream genes: p-Akt, total Akt, p-rS6, total rS6, p-4EBP1, and total 4EBP1 were evaluated to test CUDC-907 drug effect on the PI3K/Akt/mTOR pathway. PR, Myc, FOXO1, HER2, p21, and H3Ace were evaluated to test CUDC-907 drug effect on HDAC activity. β-actin was used as a loading control. **(B)** Oncogenes (Survivin, HER2, and Myc), PR downstream genes and tumor suppressor genes (FOXO1 and p21), and apoptosis markers (PARP and caspase 3 cleavage) were evaluated to test broader antitumor effects of CUDC-907. **(C)** Expression of p-Akt and PR was further validated. Caspase 8 cleavage was evaluated to test the effect of CUDC-907 on the extrinsic apoptotic pathway.

To further investigate the efficacy of CUDC-907, we first compared CUDC-907 obtained from two different resources: Selleck and MCE. As shown in **Supplementary Figure 1**, CUDC-907 from both resources inhibited the PI3K/Akt/mTOR at 1 hour and HDAC activity at 17 hours. Next, we acquired CUDC-907 from Curis and confirmed its efficacy by assessing genes critical for tumor growth over different time points. As shown in **Figure 2A**, p-Akt inhibition was observed as early as one hour after CUDC-907 treatment in Ishikawa, ECC1, and KLE cell lines. ECC1 and KLE cells also showed immediate inhibition of p-rS6, while p-4EBP1 was downregulated in both Ishikawa and KLE cells. After 17 hours of treatment, all three cell lines showed marked upregulation of PR, while the oncogenes Myc and Her2 were downregulated. Furthermore, p21 and H3Ace were upregulated in all three cell lines, while FOXO1 upregulation was observed in Ishikawa and KLE cells (**Figure 2A**).

Similar effects were observed following longer courses of CUDC-907 treatment and in an additional cell line, Hec50. As depicted in **Figure 2B**, Myc and Her2 were downregulated in all four cell lines (ECC1, Ishikawa, KLE, and Hec50). Survivin, a key anti-apoptotic protein, was most significantly downregulated in Ishikawa and Hec50 cells. Tumor suppressor genes FOXO1 and p21 were markedly upregulated in all four cell lines. Notably, 48-hour treatment with CUDC-907 resulted in the strongest upregulation of FOXO1 and p21 in ECC1, Ishikawa, and KLE cells, while the greatest effect in Hec50 cells was observed after 24 hours (**Figure 2B**).

As shown in **Figure 2C**, p-Akt was inhibited after a one-hour treatment with 1μM CUDC-907 in all four cell lines. A 24-hour treatment with 100nM CUDC-907 also downregulated p-Akt in ECC1, Ishikawa, and Hec50 cells, with 48-hour treatment showing the most notable downregulation in Ishikawa and Hec50 cells. PR was most strongly upregulated in ECC1 and Ishikawa cells following a 48-hour course of 100nM treatment, and in KLE cells after 24 hours. PR expression was not induced in Hec50 cells (**Figure 2C**), likely due to permanent PR silencing via DNA methylation, as previously reported (23, 24).

CUDC-907 also induced apoptosis; PARP cleavage, an apoptosis indicator, was observed in all four cell lines post-treatment. Additionally, CUDC‐907 treatment led to caspase 3 cleavage through the intrinsic apoptotic pathway in ECC1 and Ishikawa cells, and caspase 8 cleavage through the extrinsic apoptotic pathway in KLE and Hec50 cells (**Figures 2B, C**). These findings suggest that CUDC-907 exerts broad antitumor effects by modulating key survival and apoptotic pathways across diverse EC cell lines.

### Progesterone and progestin synergize with CUDC-907 i*n* inhibiting EC cell proliferation

As PR expression was upregulated by CUDC-907 in three EC cell lines, we tested whether progesterone (P4) or progestin (MPA) would enhance its antiproliferative effect. As shown in **Supplementary Figure 2**, MPA strongly synergized with CUDC-907 in inhibiting Ishikawa cell proliferation (Synergy score =20.44), with a Most Synergistic Area Score of 29.20, indicating a synergistic interaction. For the combination of CUDC-907 and P4, which produced a Synergy Score of 14.09 and a Most Synergistic Area Score of 28.26, suggesting a synergistic effect in specific dose regions. These results support the potential of combining CUDC-907 with hormonal therapy to enhance therapeutic efficacy in PR-positive EC.

### CUDC907 inhibits tumor growth *in vivo*

**Figure 3:**
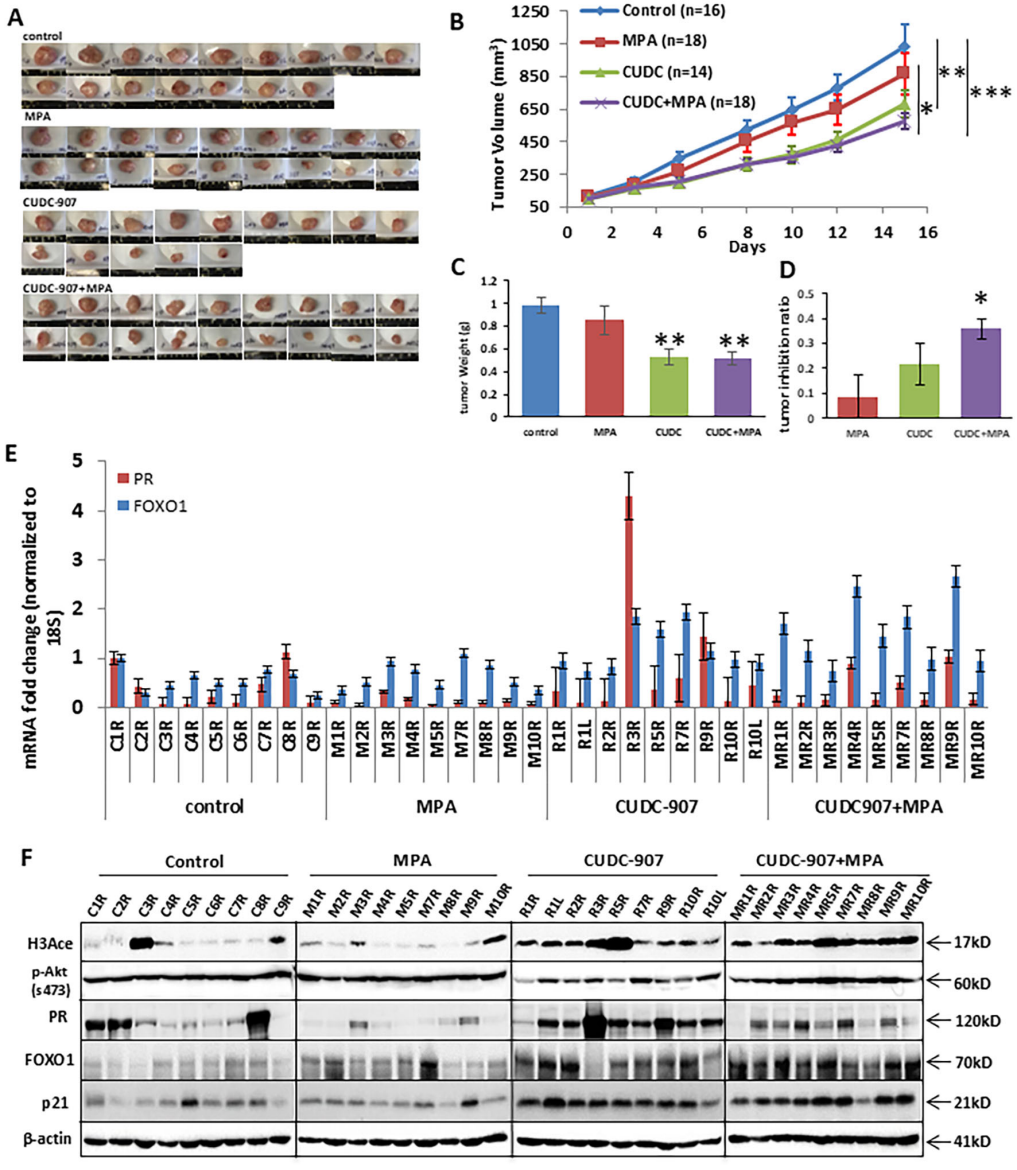
CUDC907 inhibits the growth of endometrial cancer *in vivo*. **(A)** Representative images of Ishikawa tumors are shown for each of the four treatment groups. **(B)** Mean tumor growth curves over the 21-day treatment period are shown, with tumor volumes on the final day presented as mean ± 95% confidence intervals (group 1: 1033.9mm^3^ [83.7-1229.9], group 2: 874.2 mm³ [662.99–1069.5], group 3: 679.2 mm³ [508.2–850.2], and group 4: 574.9 mm³ [464.86–684.89]) and final tumor weights for each group are shown **(C)** group 1: 0.98g [0.85-1.11], group 2: 0.85g [0.65-1.05], group 3: 0.53g [0.39-0.67], group 4: 0.51g [0.39-0.64]) are summarized. The tumor inhibition ratio represented for each group is shown **(D)** group 1: 9.40% [0%-32%], group 2: 22.32% [1%-34%], group 3: 40.47% [27%-54%] and group 4: 49.61% [42%-57%]). **(E)** mRNA expression of PR and FOXO1 was analyzed in each mouse. (* p < 0.05; ** p < 0.01, ***p<0.001) **(F)** Protein expressions of H3Ace, p-Akt, PR, FOXO1, and p21 were analyzed in each mouse. β-actin was used as a loading control.

Progesterone receptor (PR) expression is a key focus of this study. PR levels are influenced not only by HDAC inhibitor treatment but also by estrogen secreted from mouse ovarian tissue, which affects PR expression. To eliminate fluctuating hormone production, all mice were ovariectomized prior to the experiment presented in **Figure 3**.

CUDC-907 treatment significantly reduced tumor volume and weight in treated mice (groups 3 and 4), compared to those that did not receive it (groups 1 and 2) (**Figures 3A-C**). MPA treatment alone did not significantly reduce tumor volume compared to controls. In contrast, both CUDC-907 alone and in combination with MPA led to more substantial tumor growth inhibition. Tumor volume and weight reduction were comparable between the CUDC-907 alone and combination groups, and both were significantly more effective than MPA alone. The combination treatment resulted in the highest tumor growth inhibition ratio (**Figure 3D**). These results demonstrate the potent antitumor activity of CUDC-907 *in vivo*, independent of hormonal therapy.

RT-PCR analysis of tumor tissues showed that CUDC-907, both alone and with MPA, effectively increased mRNA expression of PR and its downstream target FOXO1 in most samples (**Figure 3E**). On the protein level, groups 3 and 4 showed elevated levels of H3Ace, PR, FOXO1, and p21, while p-Akt levels were markedly reduced in group 3 compared to groups 1 and 2 (**Figure 3F**). These molecular changes support the dual mechanism of CUDC-907 in restoring PR signaling and inhibiting PI3K/Akt activity *in vivo*.

### CUDC-907 alters tumor morphology and modulates HDAC- and PI3K-downstream genes expression

**Figure 4:**
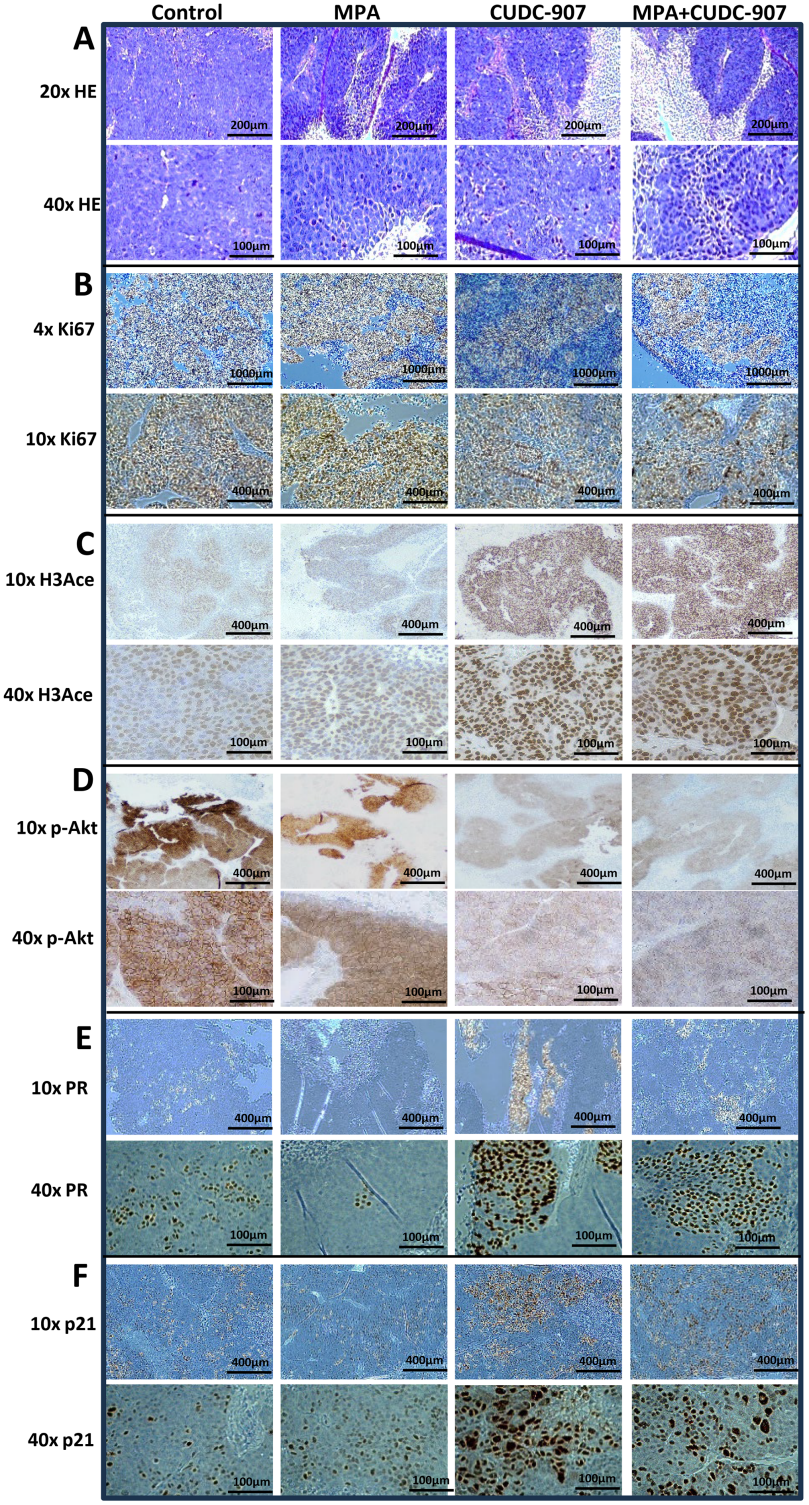
Alteration of morphology and expression of HDAC and PI3K downstream genes in tumors by H&E or IHC staining **(A, B)** H&E staining **(A)** and IHC of Ki-67 **(B)**. **(C-F)** Alteration of HDAC and PI3K pathways and PR expression in tumors by IHC staining of H3Ace **(C)**, p-Akt **(D)**, PR **(E)**, and p21 **(F)**.

As shown in **Figure 4A**, MPA treatment promoted tumor differentiation and necrosis compared to control tissue, especially in groups 2 and 4, where some neoplastic cell nests with peripheral stroma had a more organized structures and gland-like formations. CUDC-907-treated tumors showed even greater differentiation and more extensive necrosis, although cells in group 3 were less organized with fewer gland-like structures. Tumor cells in groups 3 and 4 appeared smaller than those in other groups, with the combination treatment producing the most pronounced differentiation and necrosis. These histological changes reflect enhanced tumor response to CUDC-907, particularly when combined with MPA.

Correspondingly, **Figure 4B** shows reduced Ki67 expression following MPA treatment, with further decreases after CUDC-907 treatment. The lowest Ki67 levels were observed in tumors treated with CUDC-907 + MPA. CUDC-907, alone or in combination with MPA, also strongly upregulated H3Ace, confirming HDAC inhibition (**Figure 4C**). While MPA treatment slightly reduced p-Akt levels, CUDC-907 alone or in combination with MPA caused more substantial reductions (**Figure 4D**). Although H-score analysis did not reach statistical significance (**Supplementary Figure 3A**), the trends support enhanced pathways inhibition.

PR expression decreased after MPA treatment but increased with CUDC-907 treatment, alone or in combination with MPA. Interestingly, CUDC-907 monotherapy upregulated PR more strongly than the combination treatment (**Figure 4E**), which was supported by H-score analysis (**Supplementary Figure 3B**). Lastly, both CUDC-907 and CUDC-907 + MPA markedly increased p21 expression compared to control and MPA groups (**Figure 4F**). These findings further support the role of CUDC-907 in restoring PR signaling and promoting cell cycle arrest *in vivo*.

### CUDC-907 prolongs the survival of mice bearing EC tumors

**Figure 5:**
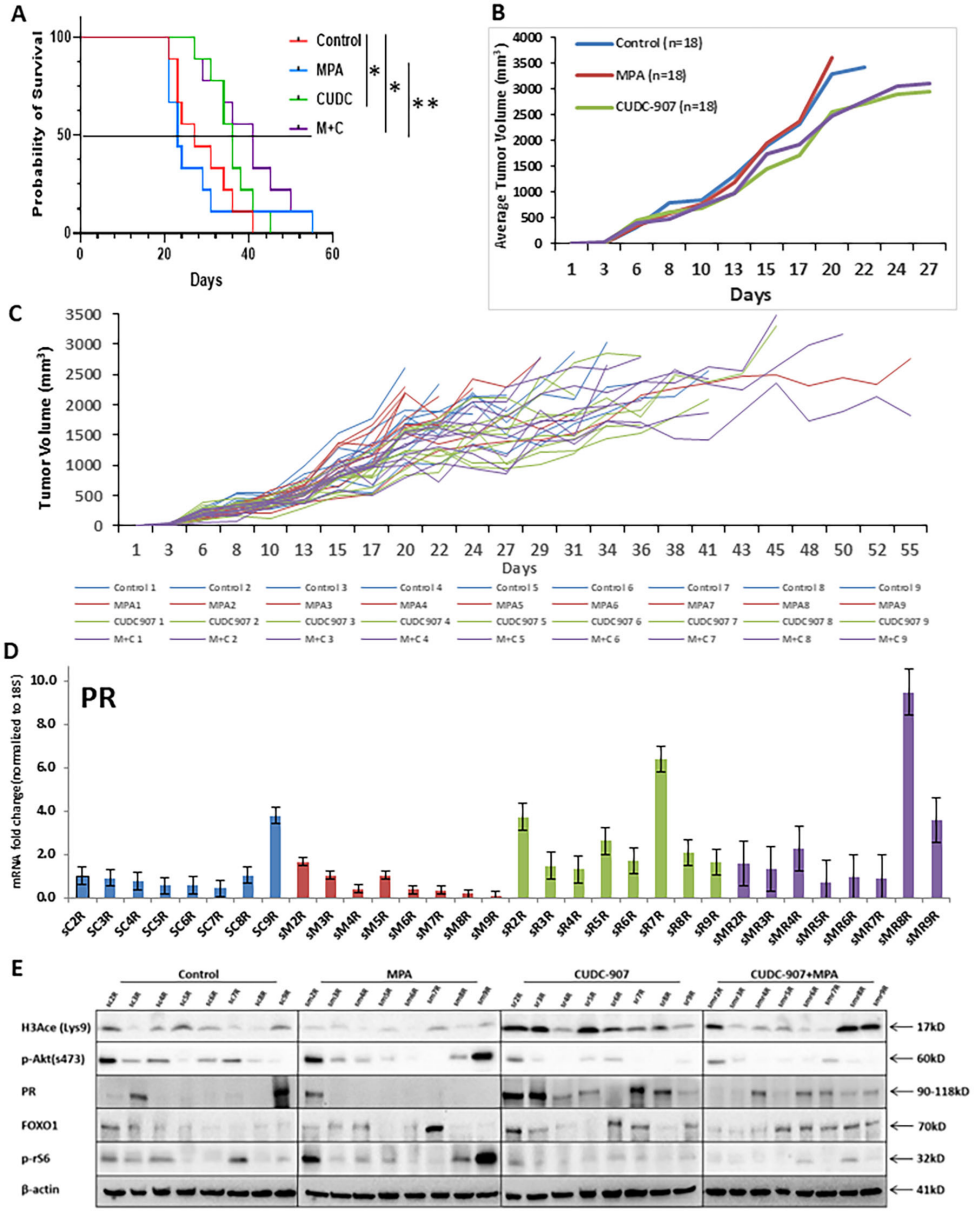
CUDC-907 prolongs the survival of mice bearing EC tumors **(A)** The Kaplan-Meier test for probability of survival and Corresponding 95% confidence intervals (CIs) were as follows: group 1: 27 days (20–35), group 2: 23 days (20–30), group 3: 36 days (26–40) and group 4: 41 days (26–49). (* p < 0.05; ** p < 0.01) **(B)** Tumor volume of the four indicated groups is summarized. **(C)** Tumor volume of each mouse was recorded up until one dimension of the tumor reached 2cm. **(D)** PR expression in each mouse was quantified. **(E)** Protein expression of H3Ace, p-Akt, PR, FOXO1, and p-rS6 were analyzed in each mouse. β-actin was used as a loading control.

To evaluate the long-term effect of CUDC-907 on survival, 45 non-ovariectomized mice were randomized into four groups. Unlike the ovariectomized model in **Figure 3**, these mice retained ovarian hormone cycling to better simulate clinical conditions. As shown in **Figure 5A**, median overall survival was extended in CUDC-907-treated and CUDC-907 + MPA groups compared to controls (OS: MPA = 22 days, control = 27 days, CUDC-907 = 30 days, CUDC-907 + MPA = 40 days). These results suggest that CUDC-907 improves survival even in hormonally intact models.

In **Figure 5B**, mean tumor volume was tracked until any tumors reached 2 cm in one dimension. CUDC-907, alone or combined with MPA, significantly reduced tumor growth compared to control and MPA alone. **Figure 5C** shows individual tumor volume trajectories. Overall, combination therapy most effectively prolonged survival, except for one outlier (MPA5) that survived 55 days.

Consistent with **Figure 3E**, mRNA expression of PR was highest in CUDC-907 treated groups (**Figure 5D**)supported by protein analysis (**Figure 5E**). PR protein levels were most strongly induced by CUDC-907 alone, while slightly reduced in the combination group, likely due to ligand-induced downregulation. H3Ace levels were strongly upregulated in both CUDC-907 groups, especially with monotherapy. p-Akt (Ser473) and p-rS6 were consistently downregulated, and FOXO1 expression increased with CUDC-907 or CUDC-907 + MPA treatment. Variability in protein expression may reflect differences in estrous cycling among mice.

### Screening of potential markers of CUDC-907 drug effect in EC tumors

**Figure 6:**
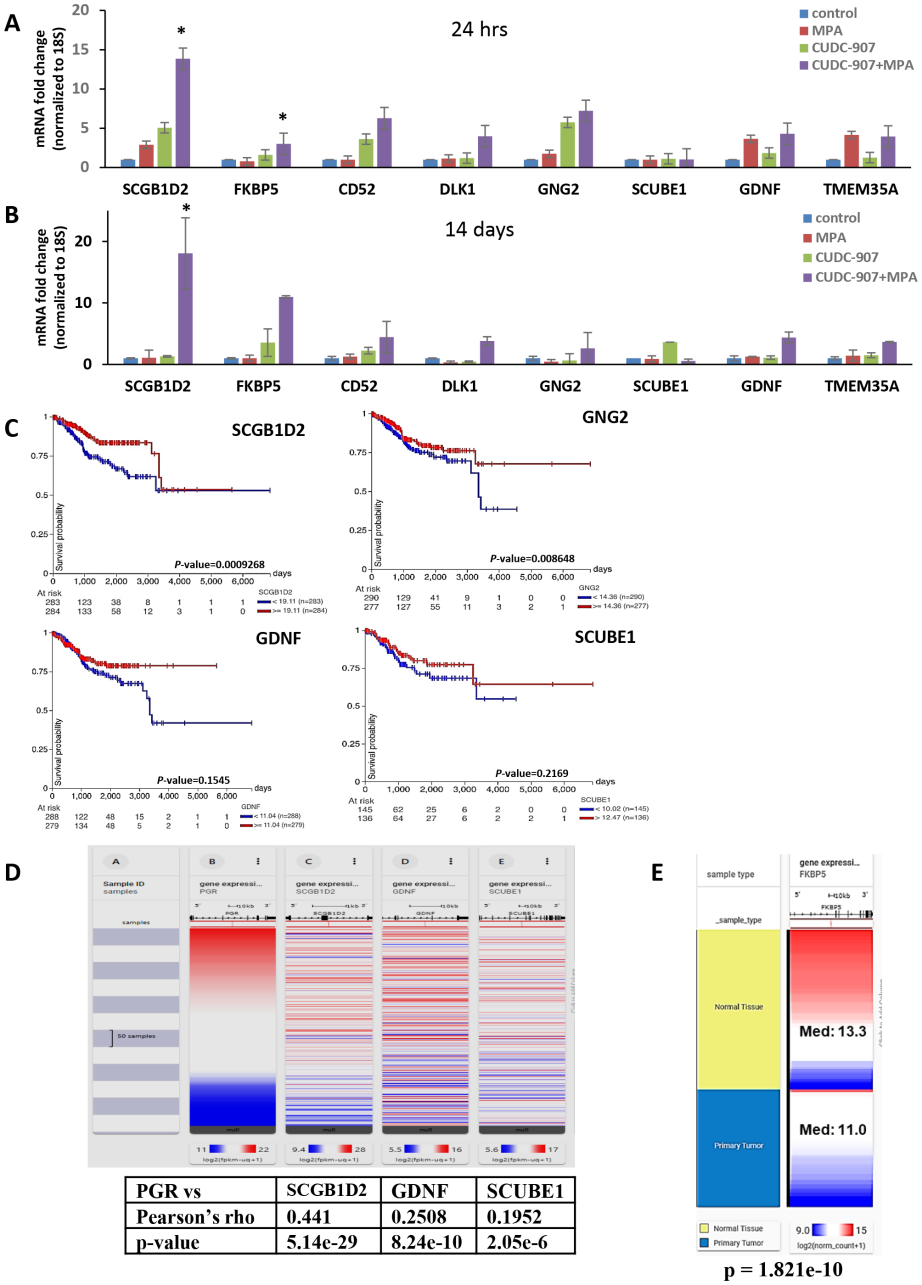
Transcriptome analysis of CUDC-907 treated Ishikawa tumors **(A)** Differentially expressed genes identified from RNA sequencing were validated via qRT-PCR over 24 hour and **(B)** 14 day treatment periods. **(C)** Kaplan Meier gene expression plots were sourced from the GDC TCGA Endometrial Cancer database using the UCSC Xena Portal. **(D)** Using the same database, of the ten genes identified, SCGB1D2, GDNF, SCUBE1, and UNC5C were observed to have positive correlations with PR (* p < 0.05). **(E)** FKBP5 expression levels in normal endometrial tissue and primary endometrial tumors from the TCGA database. Median expression was significantly higher in normal tissue (Med=13.3) compared to primary tumors (Med=11.0) (p=1.82x10^-10^)

Transcriptome analysis was conducted to identify potential markers of drugresponse(**Figures 6A, B**). Genes with reported anti-tumor functions, including *SCGB1D2 (*Secretoglobin Family 1D Member 2, *lipophilin B), FKBP5, CD52, DLK1, GALNT9, GNG2, GDNF*, and *TMEM35A*, were significantly upregulated after CUDC-907 + MPA treatment and validated by RT-PCR. Among these, SCGB1D2 showed the strongest induction (13.8-fold at 24 hours and 18.1-fold at 14 days). FKBP5 and CD52 were also robustly upregulated. Other genes, such as GNG2 and GDNF, showed consistent upregulation. Notably, several of these genes are known to modulate tumor growth and hormone signaling pathways. These genes may serve as transcriptional biomarkers of CUDC-907 response.

Kaplan Meier plots (**Figure 6C**) using TCGA Endometrial Cancer database revealed that high mRNA expression of *SCGB1D2, CD52, GNG2*, *GDNF*, and *SCUBE1*correlated with favorable prognosis in EC patients (n=547). However, p-values for *GDNF* and *SCUBE1* did not reach statistical significance. Additionally, *SCGB1D2, GDNF*, and *SCUBE1*exhibited positive correlation with PR expression (**Figure 6D**), suggesting a potential link between these markers and dual treatment responsiveness.

### IGF-1 as a potential surrogate serum biomarker for EC

**Figure 7:**
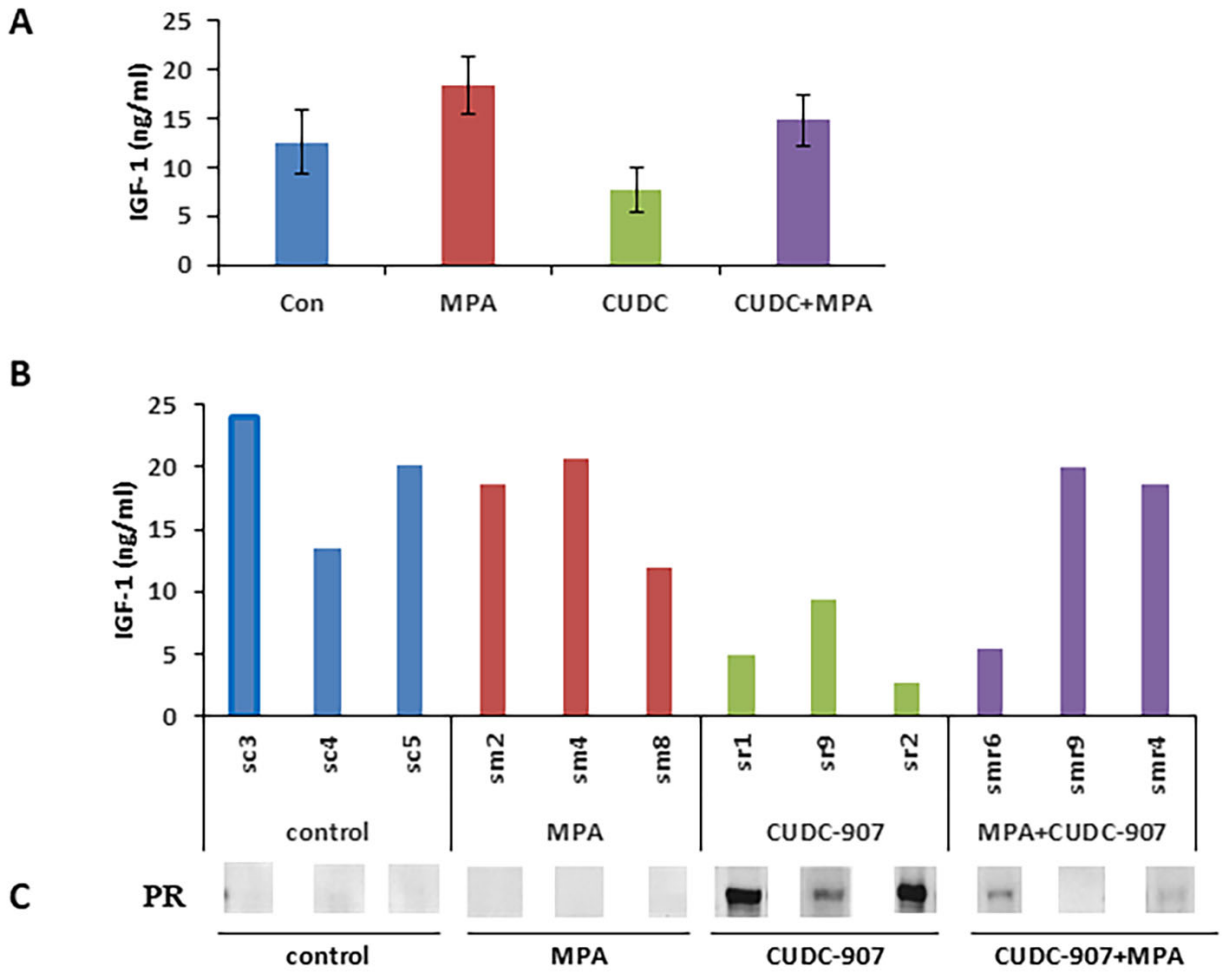
IGF-1 is a potential surrogate serum biomarker for EC **(A)** Eight cytokines from mouse serum samples were quantified via ELISA. **(B)** Circulating IGF-1 levels for each of the four indicated groups are summarized. **(C)** Correlation of circulating IGF-1 and PR protein in the tested mice is summarized.

Surrogate serum markers of drug effect were screened using a mouse cytokine ELISA kit. Eight cytokines, including leptin, TNFα, IGF-1, IL-6, insulin, IL-1α, IL-1β, and MCP-1, were quantified using the mice serum in each treatment group (**Figure 7A** and **Supplementary Figure 4**). Among these, IGF-1 levels were significantly decreased following CUDC-907 treatment. In contrast, MPA treatment increased IGF-1 levels compared to the control group (**Figure 7B**). Importantly, IGF-1 expression was inversely correlated with PR expression (**Figure 7C**). These results suggest that serum IGF-1 may serve as a non-invasive surrogate marker of CUDC-907 activity.

### CUDC-907 inhibits obesity-driven EC and reduces serum IGF-1 levels

**Figure 8:**
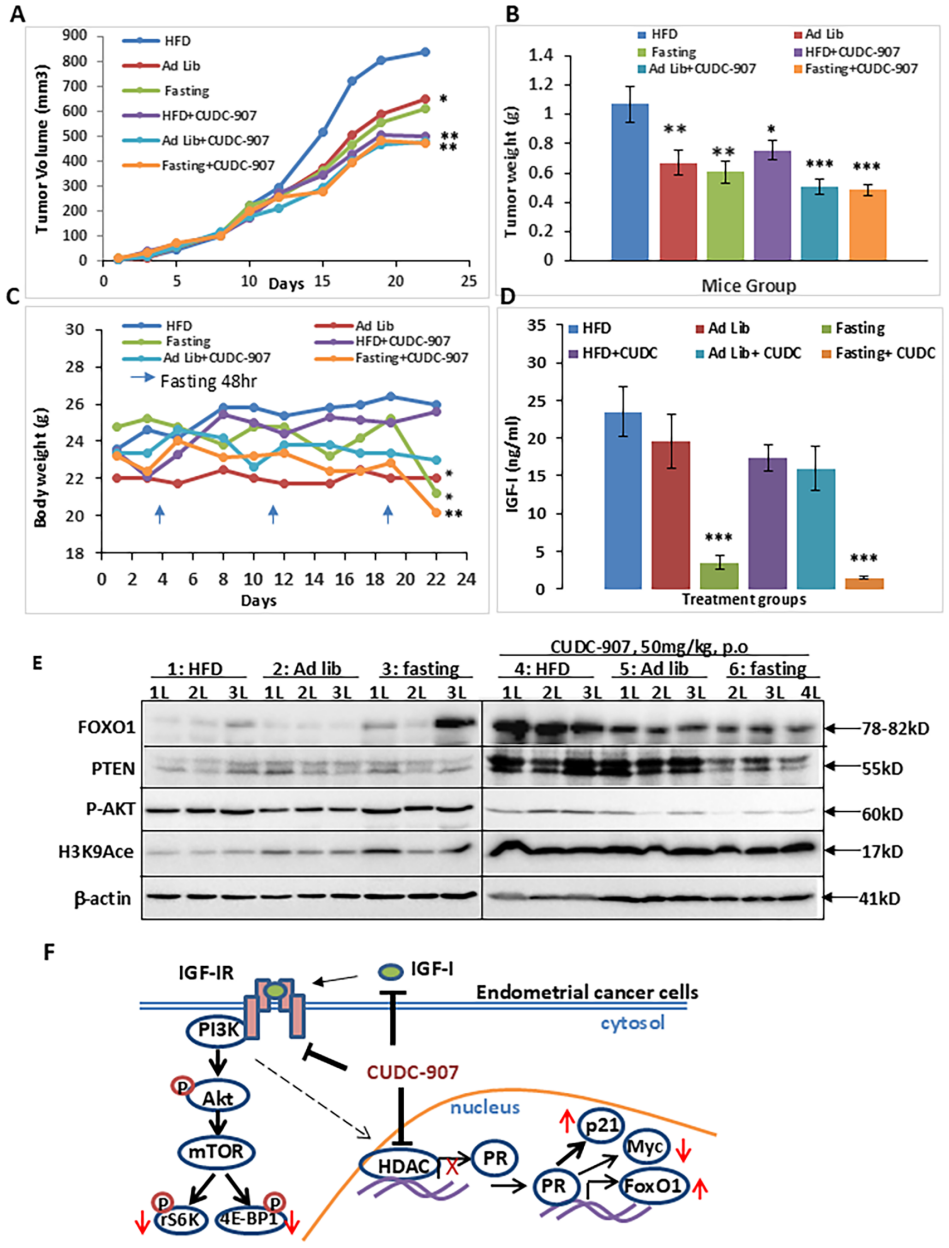
CUDC-907 decreases obesity-driven tumor growth and lowers serum IGF-1 levels. **(A)** The tumor volume on Day 22, the mean tumor sizes and their corresponding 95% CIs were as follows: group 1: 933.75 [735.08-1132.42], group 2: 649.58 [543.47-755.70], group 3: 609.07 [489.15-728.99], group 4: 502.67 [423.98-581.36], group 5:480.25 [414.00-546.51] and group 6: 493.63 [417.20-570.06]. **(B)** The mean tumor weight and their corresponding 95% CIs were as follows: group 1, 1.07g[0.83-1.31], group 2: 0.67g [0.50-0.84], group 3: 0.61g [0.46-0.76], group 4:0.75g [0.63-0.88], group 5: 0.51g[0.4-0.61] and group 6: 0.54g [0.44-0.63].**(C)** Mice body weight and their corresponding 95% CIs were as follows: group 1: 26g [23.44-28.56], group 2: 22g [21.2-22.8], group 3: 21.2g [19.13-23.27], group 4: 25.57g [24.54-26.61], group 5: 23g [20.85-25.15] and group 6: 20.43g [19.23-21.63]. **(D)** Serum IGF-1 level at the endpoint and their corresponding 95% CIs were as follows: group1: 23.45ng/ml [16.95-29.95], group 2: 19.56ng/ml [12.41-26.71], group 3: 3.45ng/ml [1.62-5.27], group 4: 17.34ng/ml [13.86-20.82], group 5: 15.99ng/ml [10.27-21.71] and group 6: 1.43ng/ml [1.09-1.77]. **(E)** Expression levels of FOXO1, PTEN, p-AKT, and Histone H3 K9acetylation were detected by Western Blotting. **(F)** Graphical summary of the main findings (* p < 0.05; ** p < 0.01, ***p<0.001).

To investigate the effect of CUDC-907 on obesity-driven EC tumor progression, mice were randomized into six groups based ib diet: high-fat diet (HFD), normal chow (ad libitum), and periodic fasting,with or without CUDC-907. Tumor growth was significantly promoted in the HFD group compared to the other groups. In all diet conditions, CUDC-907 treatment effectively reduced tumor volume to similar levels. Tumor weight was significantly reduced in the HFD group (**Figures 8A, B**). Mice treated with CUDC-907 showed slight reductions in body weight (**Figure 8C**). Serum IGF-1 levels were reduced in all CUDC-907-treated groups, with the lowest levels observed in the fasting group (**Figure 8D**). These findings highlight the potential of CUDC-907 to counteract obesity-driven EC progression.

### Summary of CUDC-907 drug effect

The dual PI3K/HDAC inhibitor CUDC-907 efficiently suppresses the PI3K/Akt/mTOR signaling pathway by decreasing the phosphorylation of Akt and mTOR downstream genes, rS6 and 4EBP1. Meanwhile, it also inhibits HDAC activity, leading to the derepression of PR expression and upregulation of well-characterized PR target genes, including p21, FOXO1, and Myc. Furthermore, CUDC-907 reduces serum IGF-1 levels. Taken together, these findings highlight CUDC-907 as a promising therapeutic strategy for EC (**Figures 8E, F**).

## Discussion

Endometrial cancer (EC) is a major gynecologic malignancy. Common treatments include surgery, chemotherapy, radiotherapy, and hormone therapy. Hormone therapy is typically used for patients with advanced (stage III or IV) or recurrent disease, or those who are ineligible for hysterectomy. However, its effectiveness often diminishes over time. Therefore, there is an urgent need to identify new therapeutic strategies and improve the efficacy of existing treatments. Furthermore, as previously mentioned, EC are unique in three aspects: (1) EC is highly sensitive to hormone regulation, (2) EC is most strongly associated with obesity among 20 cancer types, and (3) the PI3K/Akt pathway is most frequently hyperactivated in EC among the twelve major cancers. Thus, therapies targeting these distinct features of EC may lead to improved clinical outcomes. This study was designed to test the hypothesis that a dual PI3K/HDAC inhibitor could simultaneously address these three drivers of EC progression. In the current study, we identified CUDC-907 as a highly effective therapeutic candidate for EC. Specifically, CUDC-907 was shown to (1) restore PR expression and resensitize tumors to hormone therapy, (2) reduce levels of the obesity-related factor IGF-1, and (3) inhibit the PI3K/Akt signaling pathway. Importantly, CUDC-907 suppressed tumor proliferation and significantly extended mice survival, supporting its potential as a promising treatment strategy for EC patients.

Although CUDC-907 has not been studied in EC, a dual HDAC/PI3K inhibition strategy has been previously investigated. Specifically, a 2013 study examined the effects of combining an HDAC inhibitor (OBP-801/YM753) with a PI3K inhibitor (LY294002) in EC cells. This dual treatment significantly upregulated Bim expression and induced a synergistic apoptotic effect in EC cells (25). In addition, this dual regimen of HDAC and PI3K inhibitors has shown promise in other cancer types, including breast and renal cancers. Dual treatment led to greater growth inhibition and pro-apoptotic activity than monotherapy alone (25, 26).

CUDC-907 was first reported as a promising dual PI3K/HDAC inhibitor in 2012. In addition to its primary targets, CUDC-907 was shown to inhibit multiple compensatory signaling pathways, including RAF, MEK, MAPK, STAT3, and upstream receptor tyrosine kinases. Notably, CUDC-907 demonstrated greater pro-apoptotic activity and more potent tumor growth inhibition than single-target drugs (10). Since then, more than twenty follow-up studies have confirmed and expanded upon these findings (27–29), further validating the therapeutic potential of CUDC-907 across various cancer types.

In our current study, we observed that CUDC-907 rapidly inhibited the PI3K/Akt pathway and two key mTOR downstream genes, p-rS6 and p-4EBP1, in Ishikawa, ECC1, and KLE cell lines (**Figure 1C** and **Figure 2A**). Consistent with previous studies, CUDC-907 downregulated oncogenes HER2, Myc, and survivin, While upregulated tumor suppressors FOXO1 and p21 in all four EC cell lines. In contrast, PR, the ultimate EC tumor suppressor, was restored in Ishikawa, ECC1, and KLE cells (**Figure 1D**, **Figure 2A**, and **Figure 2C**). In Hec50 cells, however, PR was not re-expressed, likely due to permanent methylation of PR promoter region (23). As previously reported by our group and others, the hypomethylating agent, 5-aza-2’-deoxycytidine, is required to demethylate the PR promoter in Hec50 cells. Despite this, CUDC-907 remained effective in Hec50 cells, inhibiting the PI3K/Akt signaling pathway, inducing apoptosis, and exhibiting broad antitumor activity. Furthermore, CUDC-907 induced apoptosis in all four cell lines, as evidenced by PARP and caspase cleavage. Specifically, activation of intrinsic apoptotic pathways was validated by caspase-3 cleavage in ECC1 and Ishikawa cells, while activation of extrinsic apoptotic pathways was validated by caspase-8 cleavage in KLE and Hec50 cells (**Figure 2B**).

CUDC-907 drug efficacy was confirmed in an *in vivo* EC tumor model. Consistent with reports in other tumor types, CUDC-907 treatment greatly inhibited tumor growth (**Figure 3A-C**). To capture PR upregulation by CUDC-907, mice were treated for two weeks, as longer treatment durations would likely induce necrosis and a reduction or eventual loss of PR expression. We hypothesize that (1) extending CUDC-907 treatment beyond two weeks may further reduce tumor growth, and (2) a higher dosage could reveal a potential synergistic effect. Although, we observed a significant synergistic effect of MPA or P4 with CUDC-907 *in vitro* (**Supplementary Figure 3**), the *in vivo* data in **Figure 3D** suggest a strong additive effect, as the combination therapy outperformed either agent alone. At the mRNA level, CUDC-907 restored expression of PR and its downstream target gene, FOXO1 (**Figure 3E**), consistent with our *in vitro* findings. More importantly, at the protein level, p-Akt was decreased in CUDC-907-treated tumors. However, this observation was less pronounced in the CUDC-907 + MPA group. We hypothesize that this finding may be attributed to the phenomenon of MPA-induced increases in IGF-1 (30). Nonetheless, IHC data demonstrated that p-Akt was significantly reduced in both CUDC-907 and CUDC-907 + MPA groups in **Figure 4D**.

To identify reliable markers of drug efficacy, immunohistochemistry (IHC) was performed. Histone H3 acetylation (H3Ace), a common marker of HDAC inhibitor activity (31), was dramatically upregulated following CUDC-907 treatment (**Figure 4C**), aligning with the effects reported for other HDAC inhibitors (32). Ki67, a well-documented proliferation marker used clinically to define prognostic categories and assess treatment response, has been associated with tumor invasiveness and suppression of apoptosis (33–35). As shown in **Figure 4B**, Ki67 expression was dramatically reduced following CUDC-907 treatment, consistent with observations in other cancer models treated with CUDC-907 (31, 36). Next, p-Akt is a strong proliferation marker that activates multiple downstream signaling pathways (37), was also markedly decreased by CUDC-907 (**Figure 4D**), in line with prior reports in other cancers types. The cyclin-dependent kinase inhibitor p21, a well-known tumor suppressor (35, 38), was robustly upregulated after CUDC-907 treatment (**Figure 4F**), again consistent with findings in other cancers (31, 39). PR expression was strongly induced by CUDC-907 (**Figure 4E**). Notably, PR-expressing cells appeared to cluster together following CUDC-907 or CUDC-907 + MPA treatment. A similar pattern of unevenly distributed, PR-rich regions, referred to as “PR+ islands” has been observed in breast cancer (40).

Several transcriptomic markers were identified that may serve as indicators of CUDC-907 efficacy in EC. Among these, SCGB1D2 (lipophilin B) stood out for its strong induction (**Figure 6A-B**) and consistent association with favorable prognosis in the EC-TCGA dataset (**Figure 6C**). Its positive correlation with PR expression further suggests it may serve as a hormone-responsive marker. Previous studies have reported that lipophilin-B expression is linked to improved outcomes in ovarian cancer (21), while decreased expression of other secretoglobin family members has been associated with poor prognosis in EC (41). Additionally, SCGB1D2 upregulation has been reported in both breast and ovarian cancers (42, 43), supporting its broader role as a potential tumor suppressor.

FKBP5/FKBP51 (FKBP Prolyl Isomerase 5), a scaffold protein belongs to the immunophilin protein family, also emerged as a promising candidate. Prior research has demonstrated that FKBP5 can inhibit cell proliferation and sensitize progestin therapy via Akt inhibition (44). These findings align with its induction following CUDC-907 treatment, suggesting a potential role in mediating hormone responsiveness and growth suppression.

CD52, a glycosylphosphatidylinositol (GPI)-anchored protein, has also been reported as a favorable prognosis marker in EC, with high expression associated with improved disease-free survival (45). Our results support this observation and suggest that CD52 may be a useful biomarker of CUDC-907 activity.

GNG2, G Protein Subunit Gamma 2, implicated as a tumor suppressor in melanomas and breast cancer (46, 47), was also upregulated in response to treatment. Notably, GNG2 is a direct transcriptional target of PR in decidualized human endometrial stromal cells (48), reinforcing its relevance as a hormone-regulated gene. Its positive prognosis association in EC further supports its potential utility as a marker of treatment response.

GDNF (Glial Cell Derived Neurotrophic Factor) presented a more complex profile. Although GDNF is frequently described as oncogenic in various cancers, including EC (49), some studies suggest it may also regulate neuronal differentiation at post-migratory stages (50) and has been implicated in the regulation of growth, differentiation, and apoptosis in neuroblastomas (51). While its expression correlated positively with PR and predicted better survival in the EC-TCGA dataset, the lack of statistical significance underscores the need for further investigation to clarify its role in EC biology. Future studies should explore whether GDNF functions as a context-dependent modulator of hormone signaling or tumor progression in EC. Together, these findings suggest that CUDC-907 modulates a set of genes with prognostic and functional relevance, some of which may serve as biomarkers of therapeutic response or as mechanistic links to hormonal signaling.

To identify potential surrogate serum markers for CUDC-907 drug efficacy, we screened eight serum cytokines using ELISA. Among the eight cytokines, Insulin-like Growth Factor 1 (IGF-1) was the most significantly altered cytokine after CUDC-907 treatment, showing a 38.4% decrease compared with the control (**Figure 7A**). Elevated circulating IGF-1 levels have been positively associated with increased risk of breast, prostate, and colorectal cancers (52–54). Although IGF-1 has not been widely studied in EC, it is well recognized that IGF-1 signaling plays a key role in EC development, particularly in patients with insulin-resistant type II diabetes (55, 56).

IGF-1 functions as a critical regulator of cell cycle progression and survival in multiple malignancies, primarily through activation of the PI3K/Akt pathway and the mitogen-activated protein kinase (MAPK), ultimately leading to the suppression of apoptosis (57). In EC specifically, IGF-1 has been reported to promote growth through the PI3K signaling axis (58). Our findings suggest that IGF-1 may serve as a useful surrogate serum biomarker of CUDC-907 response, as its levels decline markedly following CUDC-907 treatment. Notably, we also observed an inverse correlation between tumor PR expression and serum IGF-1 concentration (**Figure 7B**). This observation suggests that serum IGF-1 l may predict PRrestoration and treatment responsiveness. This observation is consistent with previous reports in breast cancer, where IGF-1 has been shown to suppress PR expression via the PI3K/Akt pathway (59). However, similar studies in EC are lacking, and further research is warranted to validate IGF-1 as a predictive biomarker in this context.

Obesity is a well-established risk factor for EC, with approximately 70% of EC patients classified as overweight. Despite this strong association, effective clinical interventions targeting obesity-driven EC remain limited, with metformin being the only agent tested in phase I clinical trials to date. Our current studies suggest that CUDC-907 reduces both tumor burden and serum IGF-1 levels in obesity-associated EC models (**Figures 8A, D**). highlighting its potential as a novel therapeutic strategy. However, additional preclinical and clinical studies are necessary to further validate these observations. In this context, serum IGF-1 could serve as a hormone-related biomarker, while circulating tumor DNA (ctDNA) may provide a complementary, tumor-specific readout for treatment response and residual disease. Recent studies have shown that ctDNA dynamics are highly sensitive for detecting recurrence and monitoring response in EC (60). The integration of IGF-1 and ctDNA as dual biomarkers may improve therapeutic monitoring and guide individualized treatment decisions in future clinical trials.

This study provides comprehensive evidence supporting the efficacy of CUDC-907 in EC, demonstrating its ability to inhibit tumor growth, restore PR expression, suppress the PI3K/Akt pathway, and reduce serum IGF-1 levels, particularly in obesity-driven disease models. A major strength of this work lies in its integrative approach, which combines *in vivo* tumor modeling, transcriptomic profiling, cytokine screening, and IHC to identify pharmacodynamic markers and elucidate mechanisms of drug action. Moreover, the identification of potential surrogate serum and tissue biomarkers, such as IGF-1, SCGB1D2, and FKBP5, adds translational value and may inform future clinical applications.

Nonetheless, several limitations should be acknowledged. Only one EC tumor model (Ishikawa) was tested *in vivo*, which may limit the generalizability of our findings. Further validation using additional EC cell lines and patient-derived xenografts (PDX) is needed. In parallel, although promising biomarker candidates were identified, their functional roles in mediating drug response were not directly tested. Lastly, while several potential drug combinations with CUDC-907 have been proposed, their efficacy in EC remains to be experimentally tested.

CUDC-907 has demonstrated broad anti-cancer activity across multiple cancer types, including prostate, bladder, esophageal cancers, as well as multiple myeloma. However, in pancreatic cancer, resistance has been attributed to compensatory activation of the mTOR and MEK/ERK pathways (61). Notably, combining CUDC-907 with the PI3k/mTOR dual inhibitor, VS-5584 has yielded superior antitumor effects in preclinical studies. In addition to VS-5584, over ten agents have been evaluated in combination with CUDC-907 to enhance its therapeutic effect. These include inhibitors targeting diverse oncogenic pathways, such as the BCL-2 inhibitor venetoclax (62), PARP inhibitor olaparib (31), Abl inhibitors ponatinib and imatinib (63), and the proteasome inhibitor carfilzomib (64). Given the encouraging outcomes from these combination regimens, it is critical to identify the most effective agents to pair with CUDC-907 and elucidate the molecular mechanisms underlying their synergy. Future studies will focus on evaluating these combinations in EC models, with the goal of optimizing therapeutic efficacy and overcoming resistance mechanisms.

Building on our preclinical findings and prior Phase 0 experience (NRG-GY011) (13), we propose a future Phase I/II clinical trial to evaluate whether CUDC-907 can enhance the efficacy of progestin therapy in patients with endometrioid endometrial cancer. CUDC-907 has demonstrated the ability to restore progesterone receptor (PR) expression, supporting its potential as a neoadjuvant or combination agent to sensitize PR-expressing tumors to progestin treatment. The proposed trial would assess the safety and biological activity of CUDC-907 in combination with medroxyprogesterone acetate (MPA), with pharmacodynamic markers (PR, FOXO1, H3K9 acetylation, pAkt, Ki67, and serum IGF-1) and histologic response as primary and secondary endpoints, respectively. While PR-positive tumors are the most apparent candidates, further investigation is warranted to determine the benefit of this approach in broader subgroups, including patients with obesity-driven disease or mismatch repair–deficient (MMR-d) tumors, which may exhibit partial hormonal responsiveness. This trial would test the novel hypothesis that dual PI3K/HDAC inhibition can restore hormone sensitivity and overcome resistance in biologically diverse EC subtypes. If successful, this study could establish CUDC-907 as a clinically actionable agent that enhances progestin therapy and expands treatment options for EC patients.

## Data Availability

The original contributions presented in the study are included in the article/**Supplementary Material**. Further inquiries can be directed to the corresponding author.
