# Supplementary material for: Dual PI3K and HDAC inhibitor, CUDC-907, effectively inhibits endometrial cancer growth in vitro and in vivo
Source: Front Oncol. 2025 Nov 4;15:1531805. doi: 10.3389/fonc.2025.1531805 (PMC12623209; doi:10.3389/fonc.2025.1531805)

Supplemental Table 1: IC50 of BKM120, LBH589 and CUDC907 In endometrial cancer cell lines.

	BKM120 (nM)	LBH589 (nM)	CUDC907 (nM)
ECC1	15	20	9.5
Ishikawa	650	18.4	9.8
KLE	250	25	35
Hec50	73	85.6	48.5

Supplemental Fig 1: CUDC-907 inhibits PI3k/Akt/mTOR signaling pathway and transcriptional inhibition of HDAC activity and increases functional PR expression

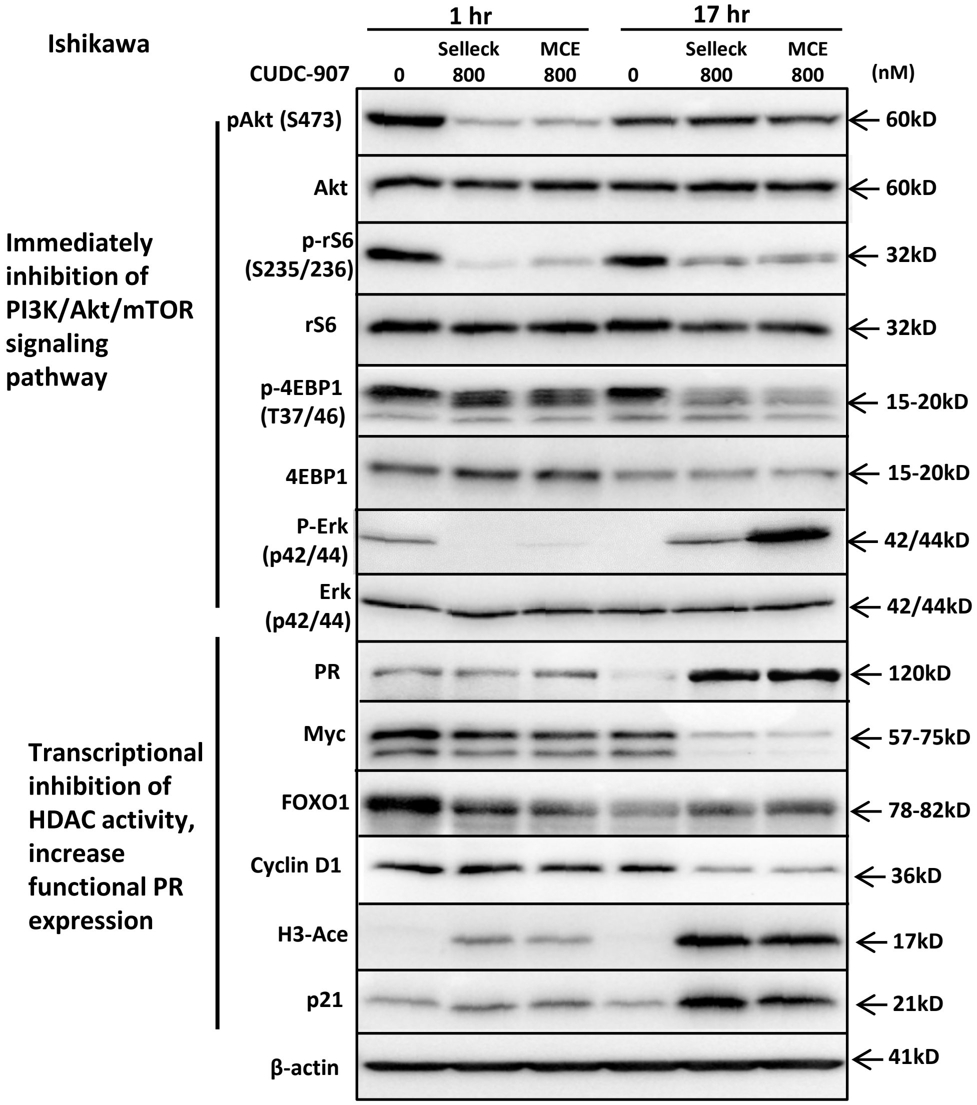

Supplemental Fig 2: CUDC-907 and progestin synergistic effect

Drug combination	Synergy score	Most synergistic area score	Method
P4+CUDC-907	14.09	28.26	ZIP
MPA+CUDC-907	20.44	29.20	ZIP

A

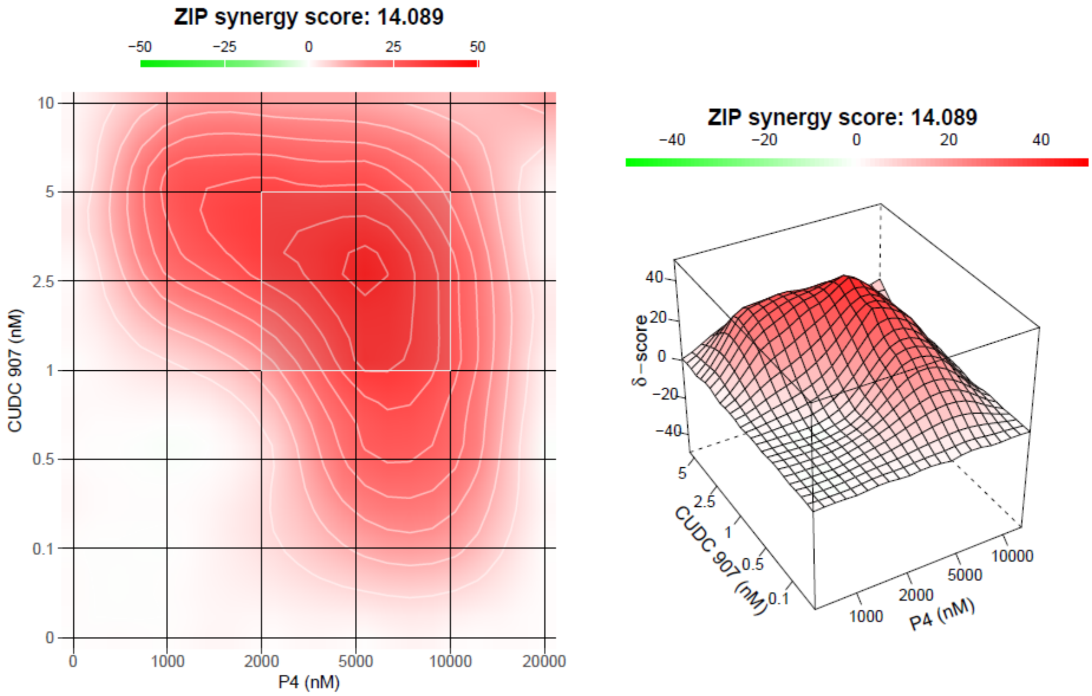

B

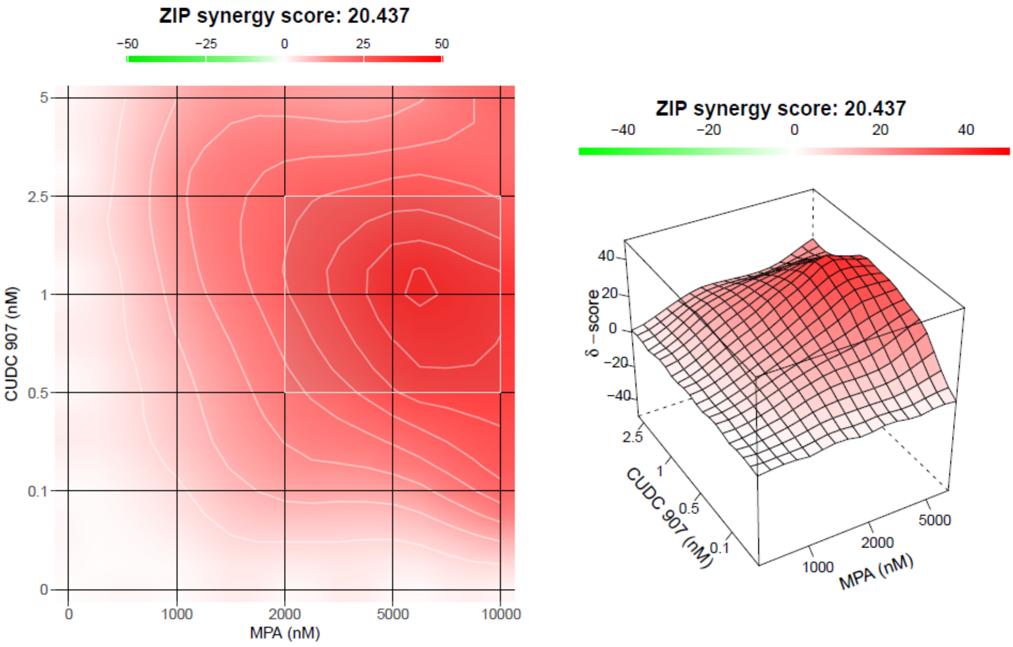

Supplemental Fig 3: IHC score of p-AKT and PR

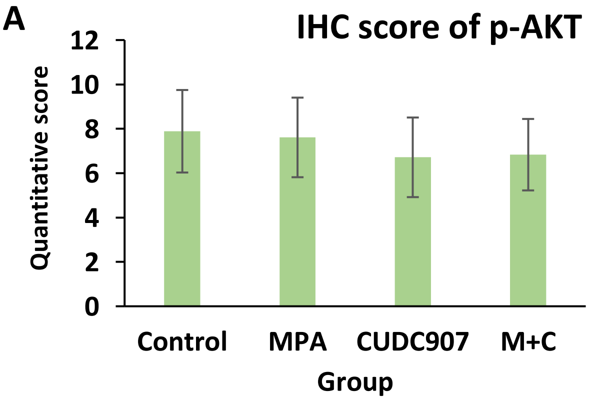

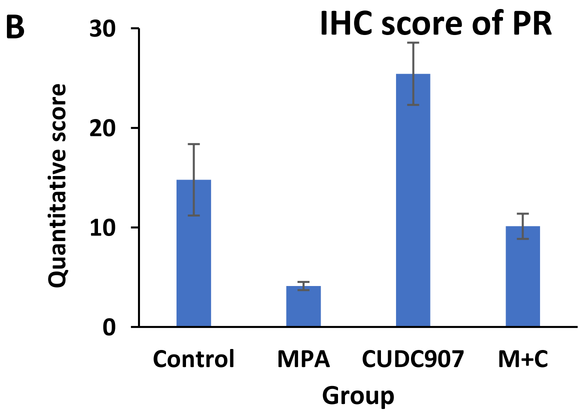

Supplemental Fig 4: circulating markers

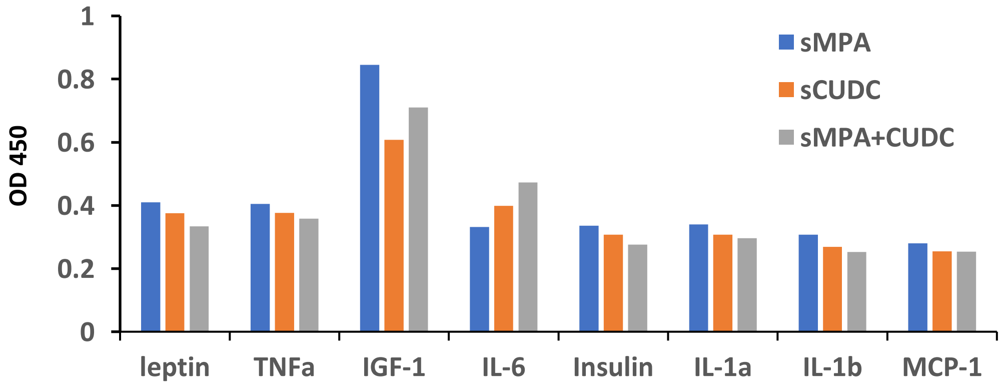

Supplemental Fig 5: Protein quantification (related to Fig.1C)

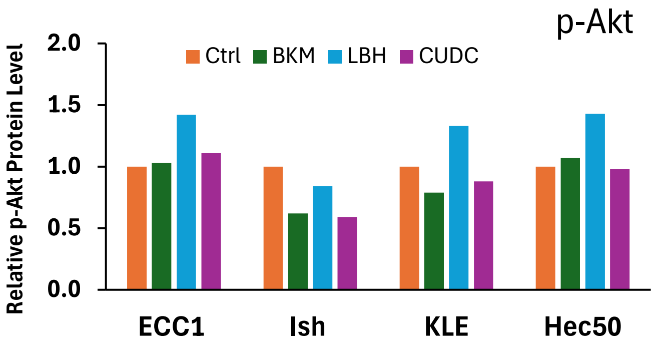

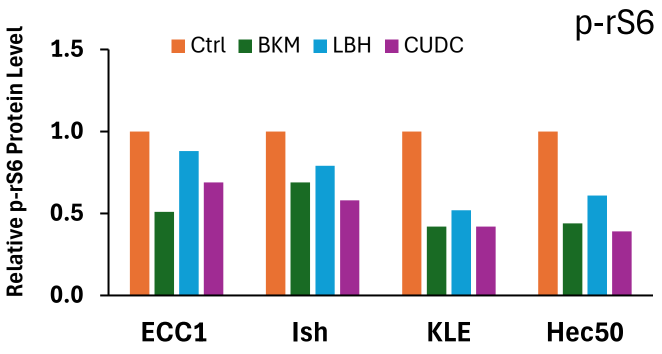

Protein quantification (related to Fig.1D)

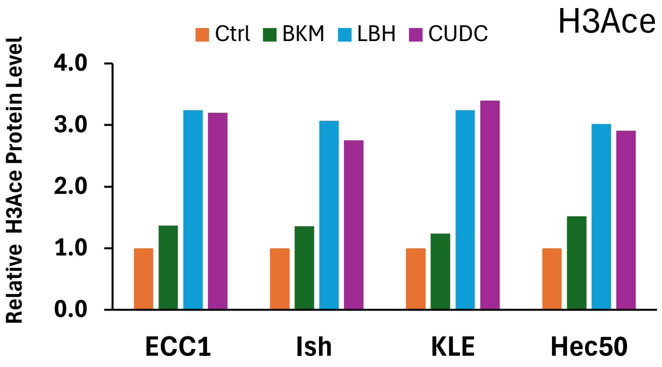

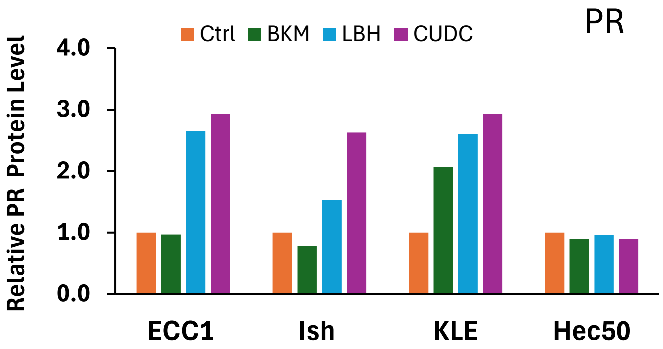

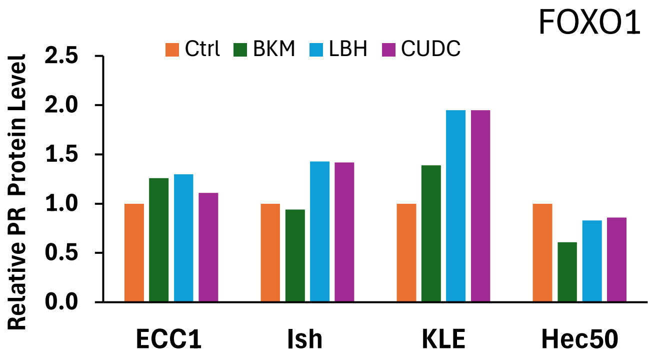

Supplemental Fig 6: Protein quantification (related to Fig. 2A)

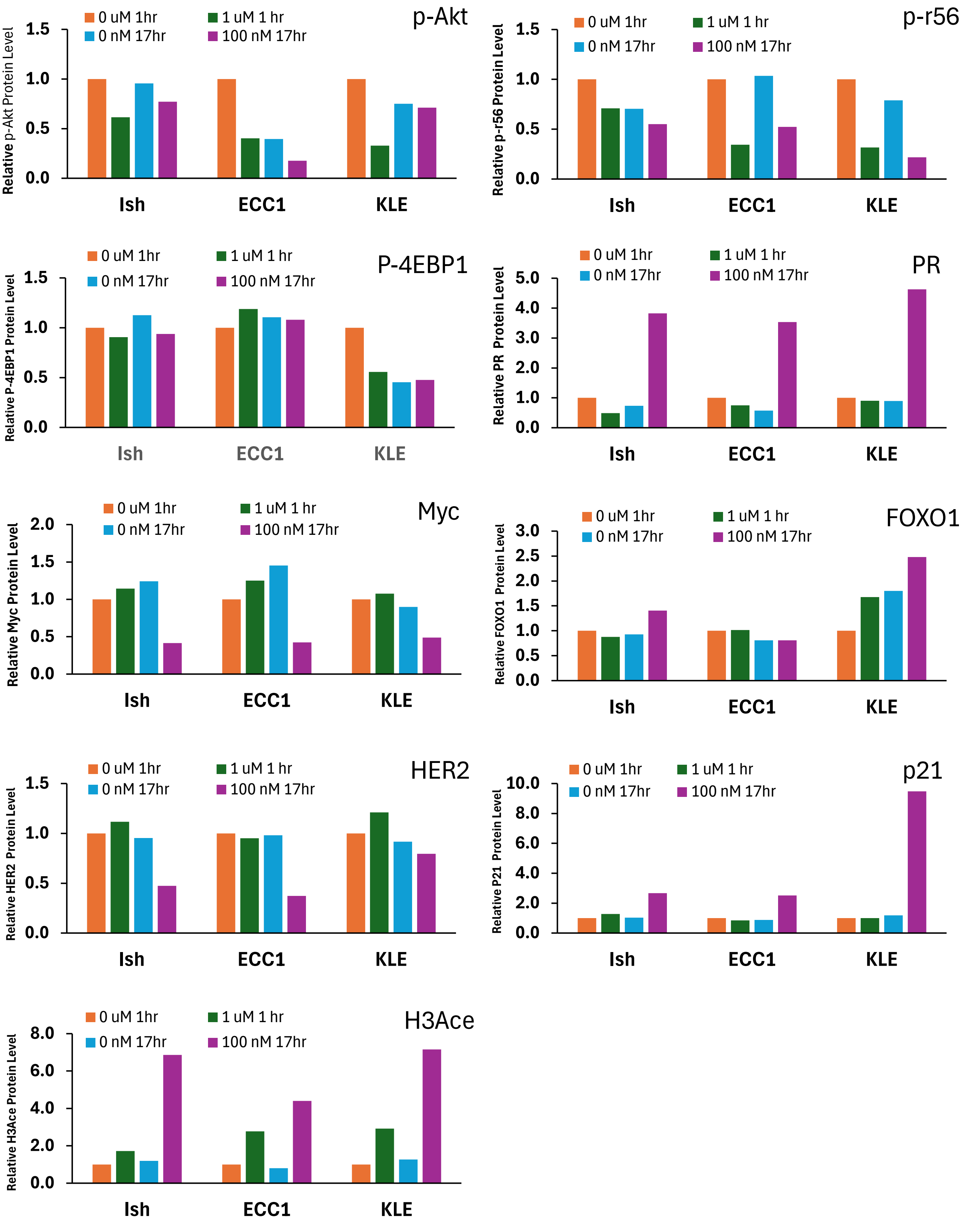

Supplemental Fig 6 (continued): Protein quantification (related to Fig. 2B)

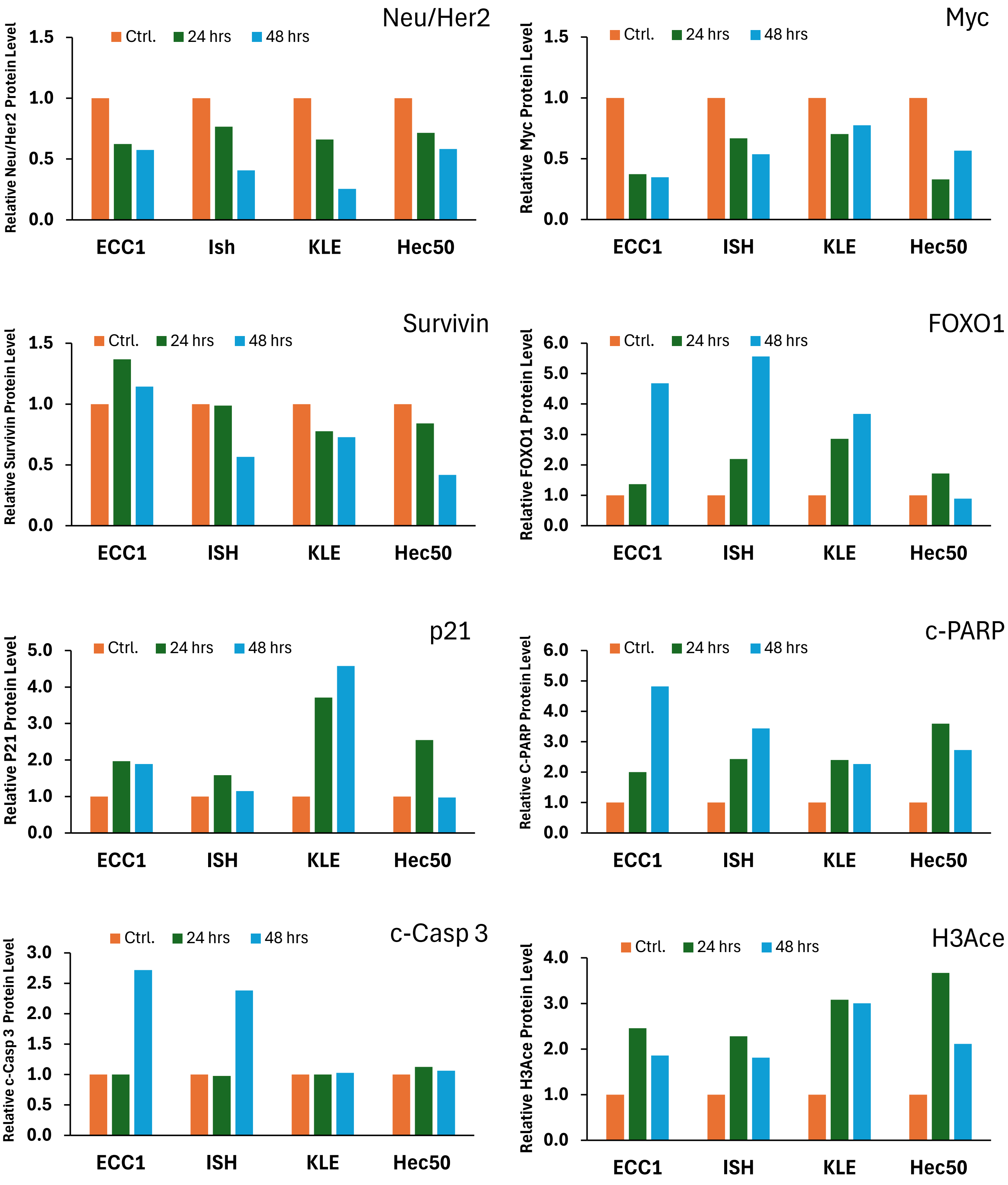

Supplemental Fig 6 (continued): Protein quantification (related to Fig. 2C)

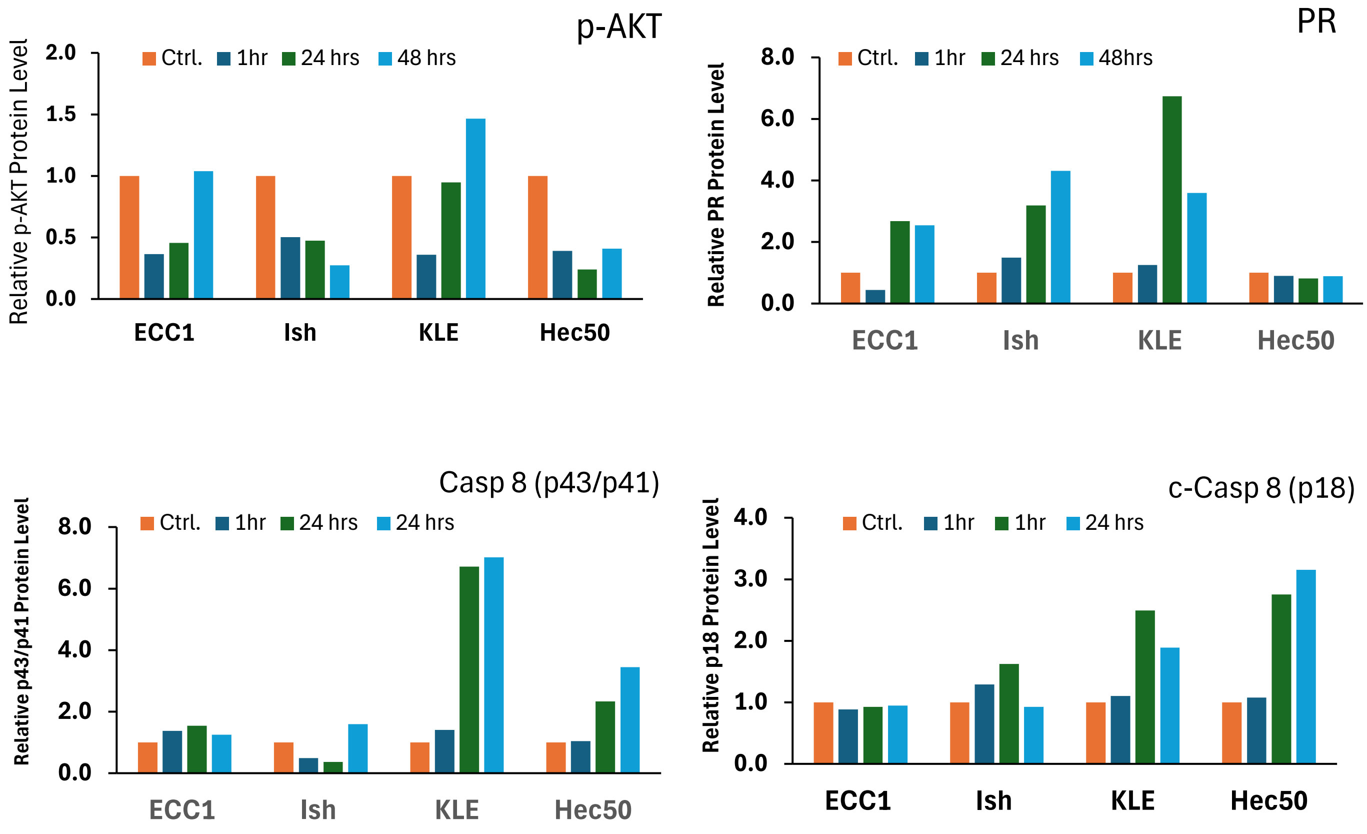

Supplemental Fig 7: Protein quantification (related to Fig. 3F)

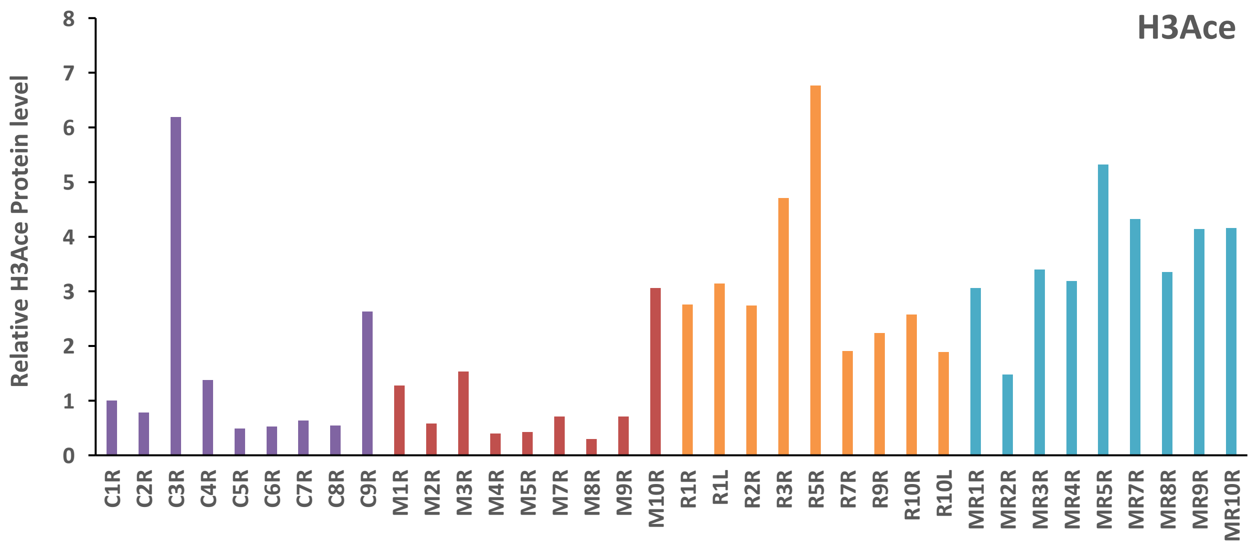

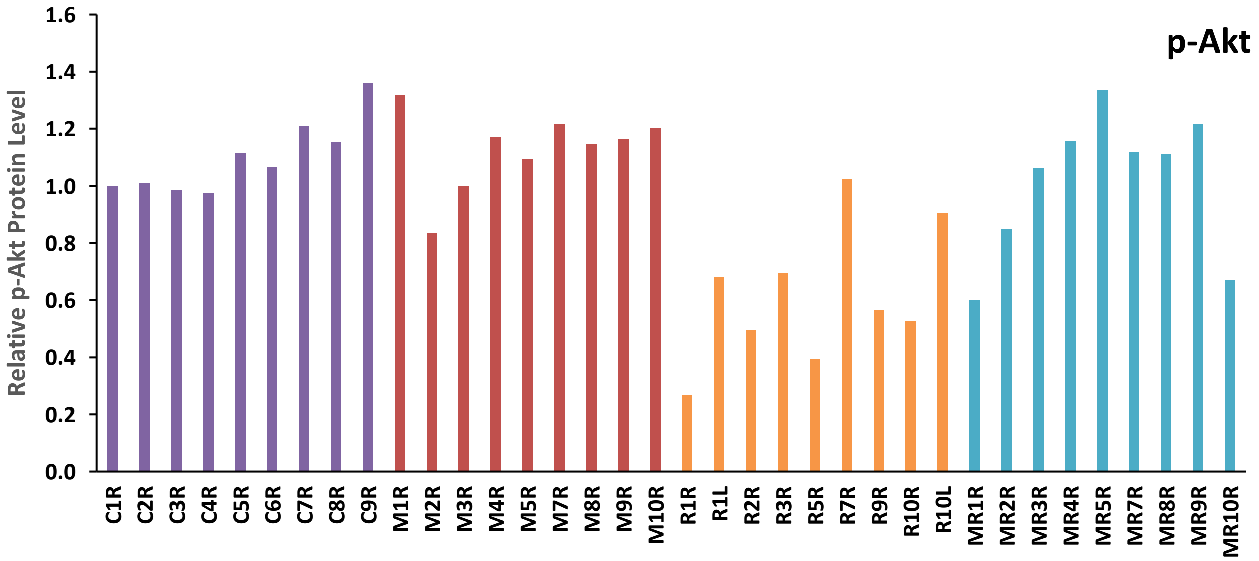

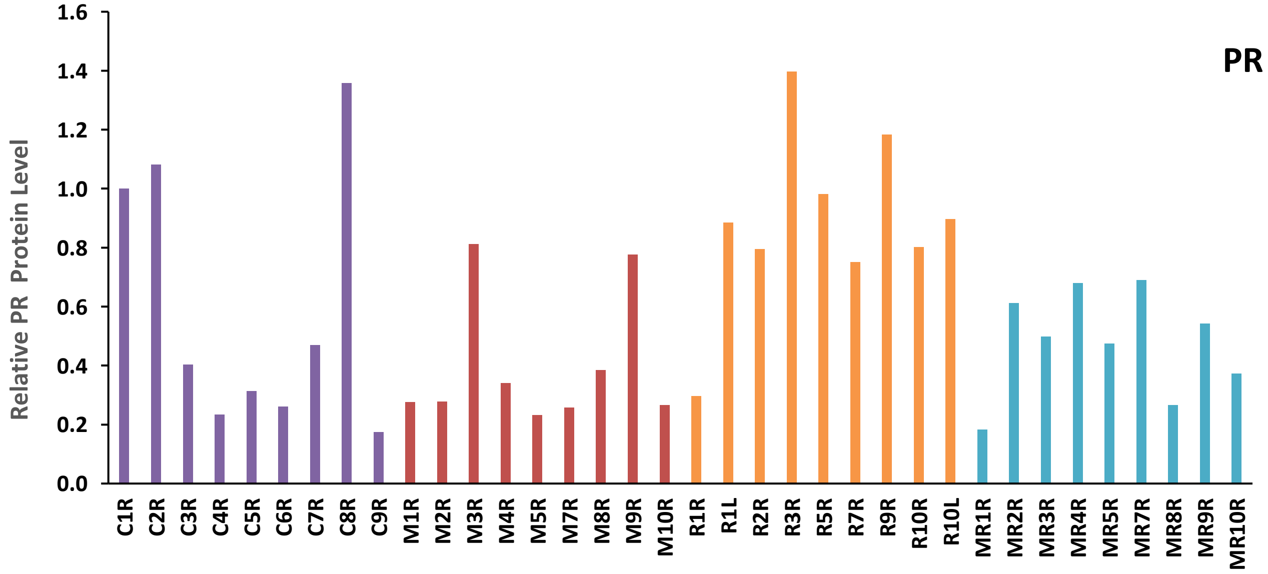

Supplemental Fig 7 (continued): Protein quantification (related to Fig. 3F)

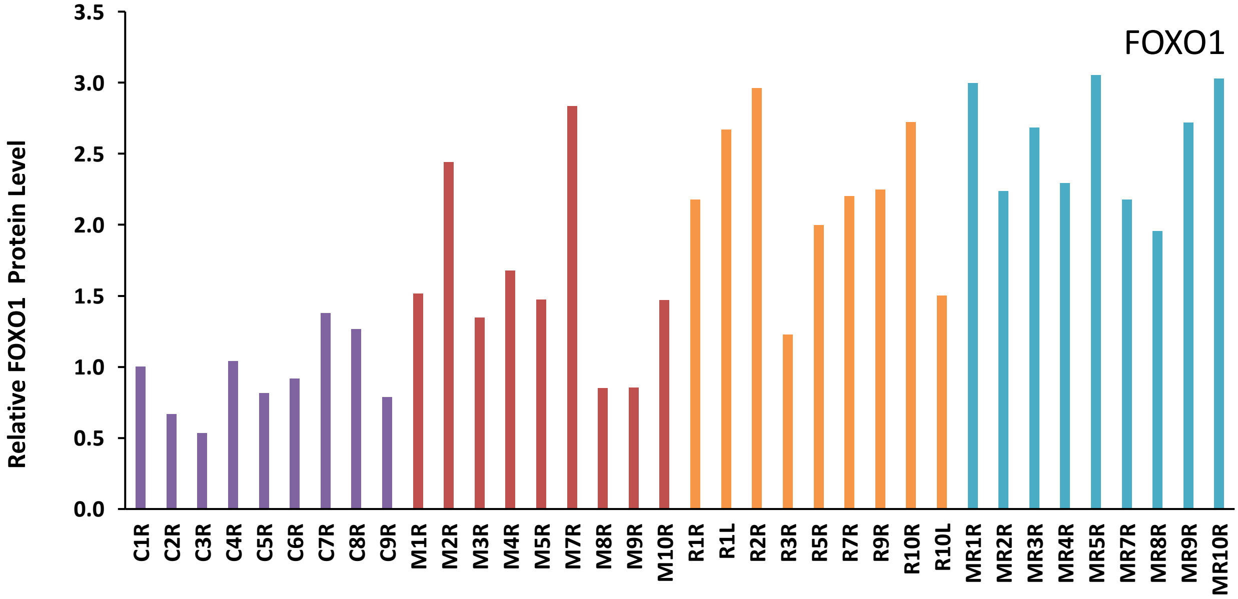

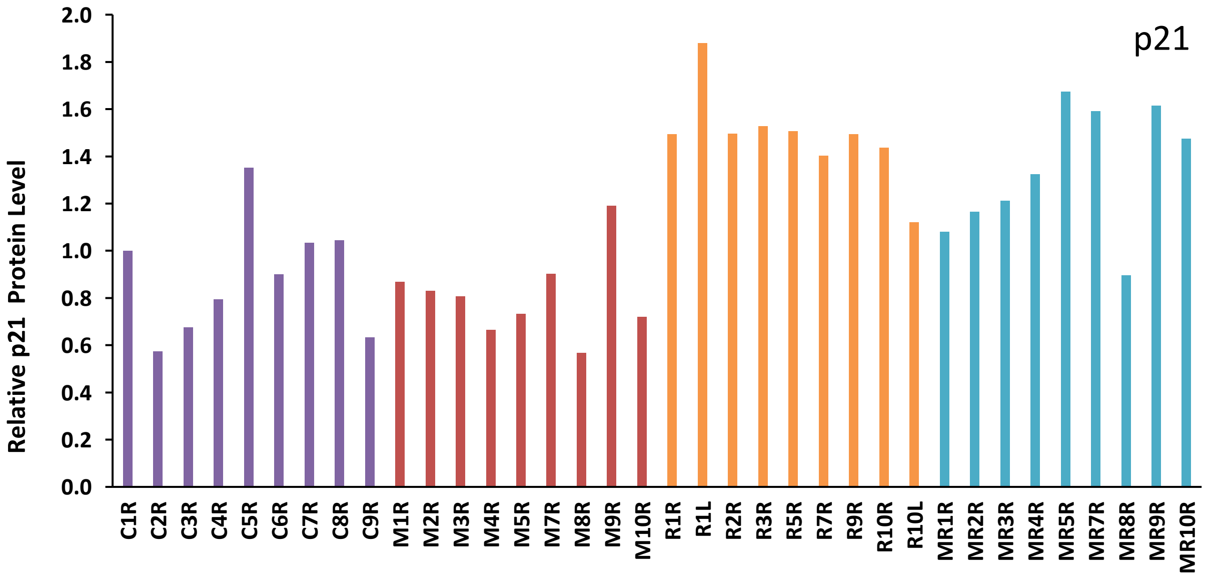

Supplemental Fig 8: Protein quantification (related to Fig. 5E)

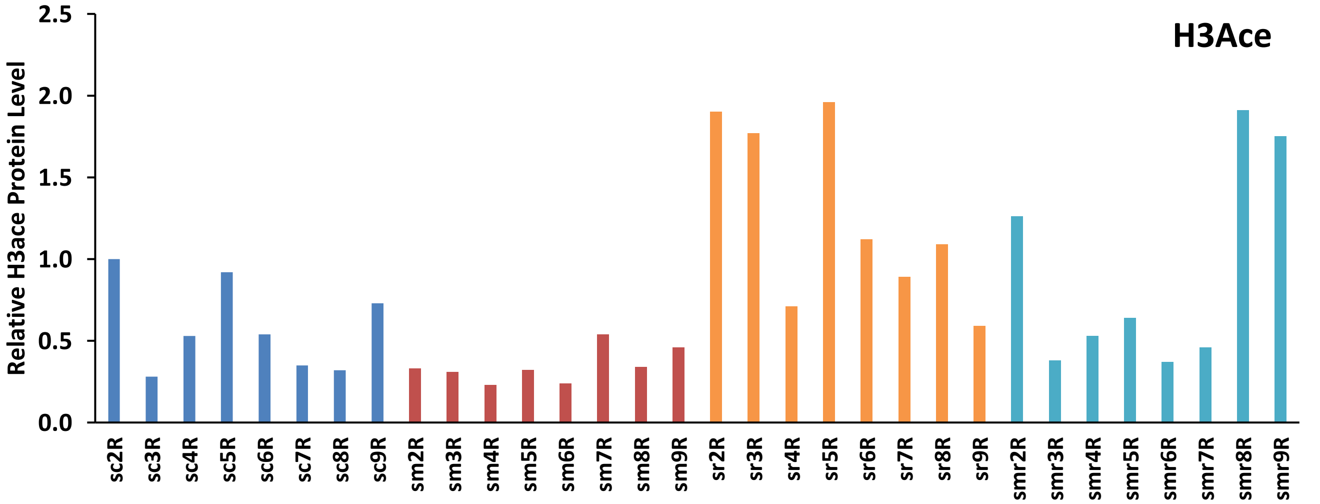

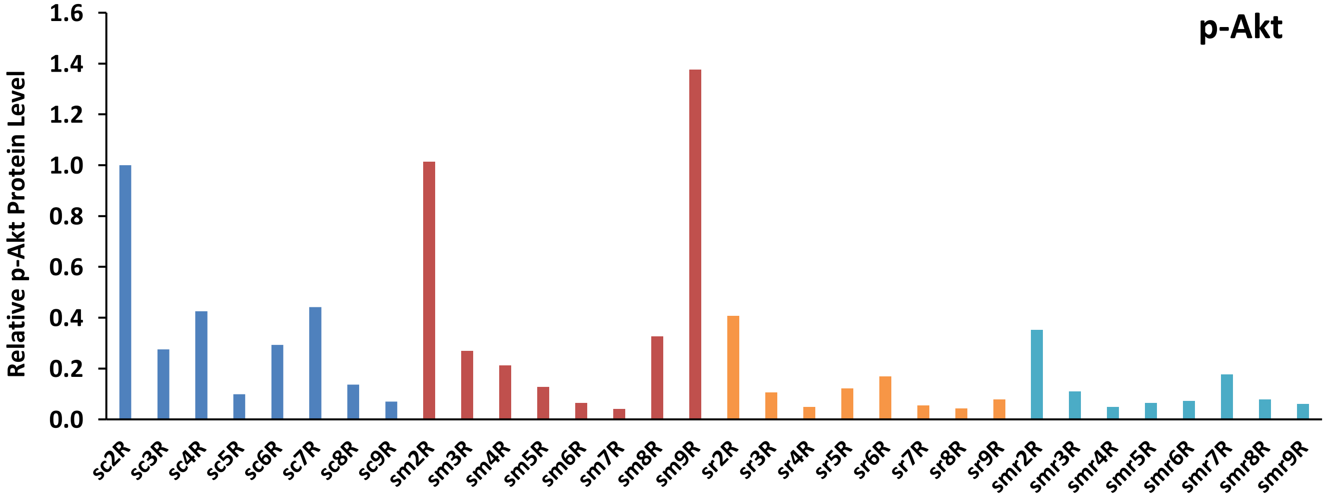

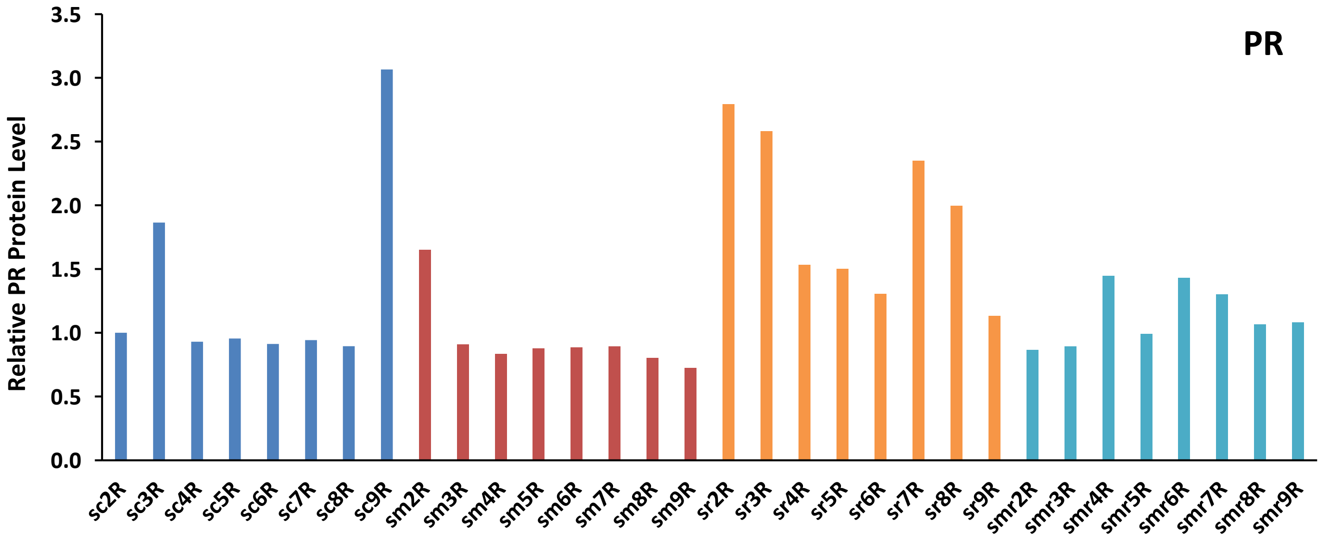

Supplemental Fig 8 (continued): Protein quantification (related to Fig. 5E)

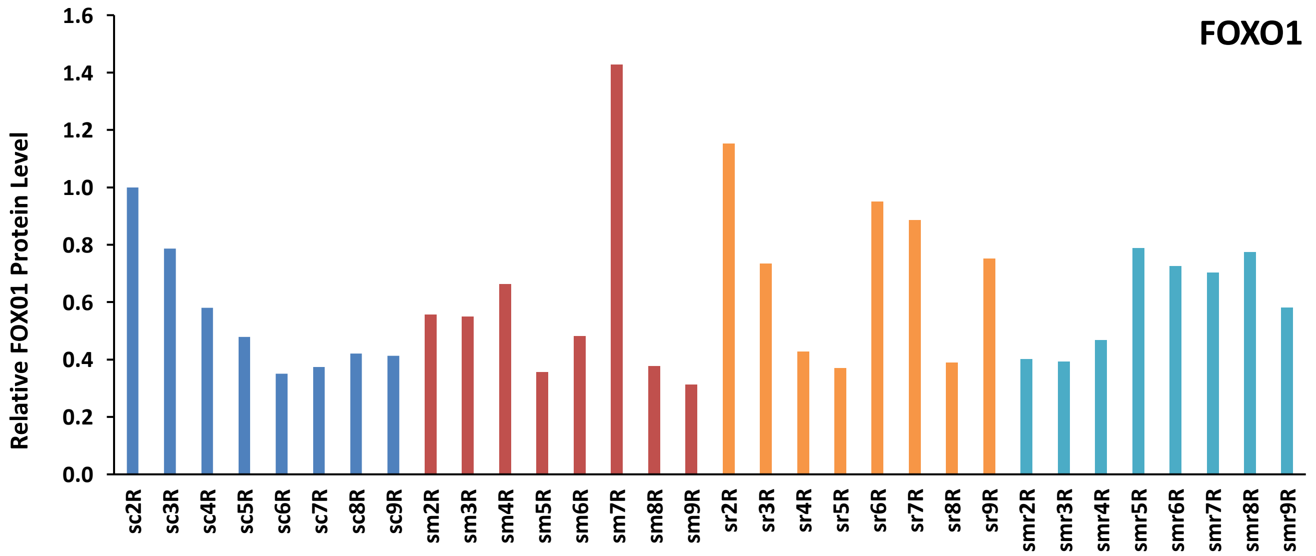

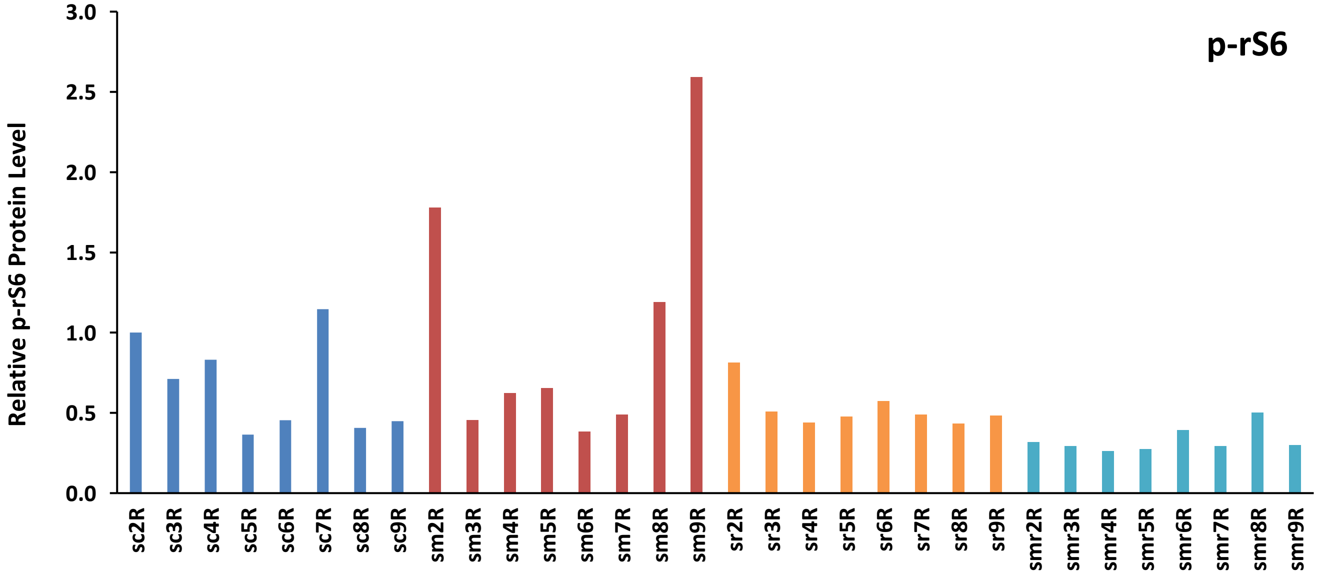

Supplemental Fig. 9: Protein quantification (related to Fig. 8E)

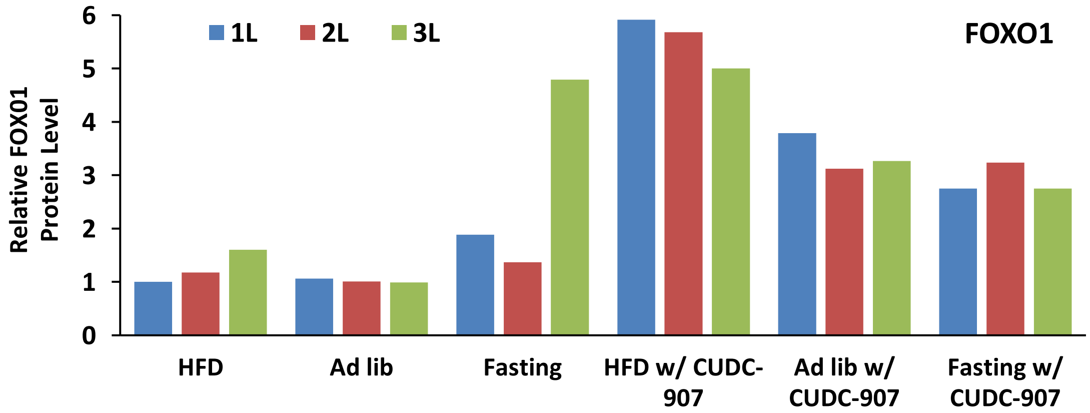

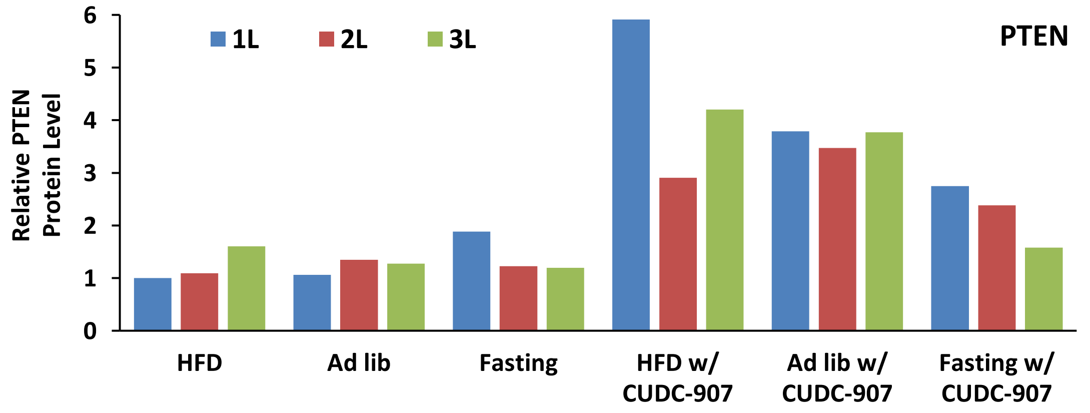

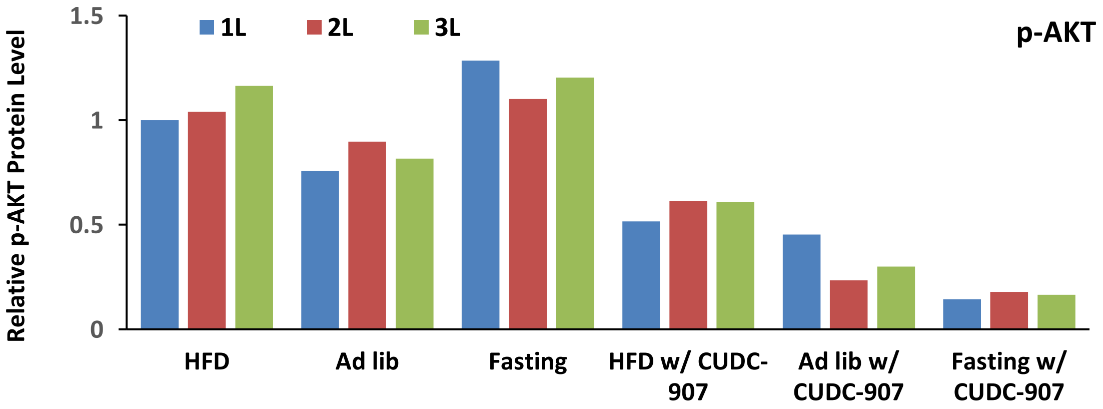

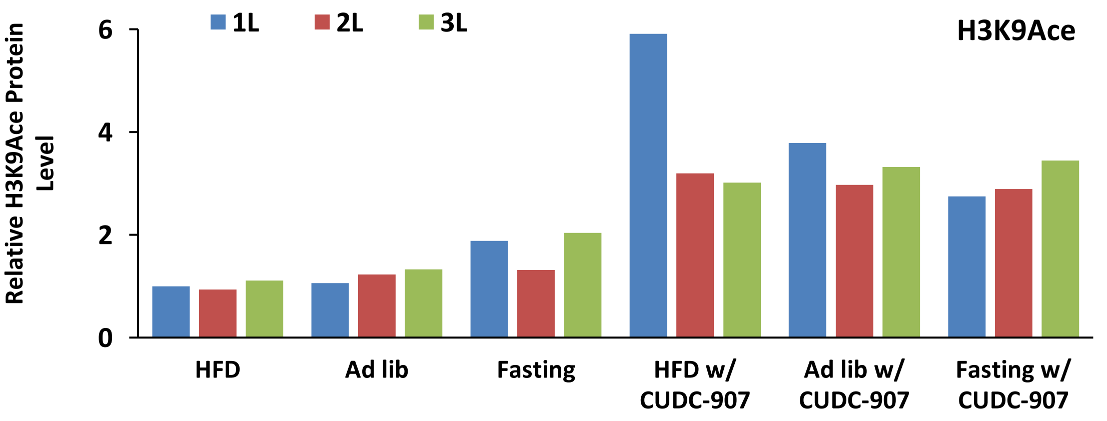

Supplement: Supplementary file 1 [file Image1.pdf]
